# Recurrent mutations at estrogen receptor binding sites alter chromatin topology and distal gene expression in breast cancer

**DOI:** 10.1186/s13059-018-1572-4

**Published:** 2018-11-07

**Authors:** Jiekun Yang, Xiaolong Wei, Turan Tufan, Cem Kuscu, Hayrunnisa Unlu, Saadia Farooq, Elif Demirtas, Bryce M. Paschal, Mazhar Adli

**Affiliations:** 1 Department of Biochemistry and Molecular Genetics, University of Virginia School of Medicine, 1340 Jefferson Park Ave, Pinn Hall, Room: 6228, Charlottesville, VA 22903 USA; 2 Center for Cell Signalling, University of Virginia School of Medicine, Charlottesville, VA USA

## Abstract

**Background:**

The mutational processes underlying non-coding cancer mutations and their biological significance in tumor evolution are poorly understood. To get better insights into the biological mechanisms of mutational processes in breast cancer, we integrate whole-genome level somatic mutations from breast cancer patients with chromatin states and transcription factor binding events.

**Results:**

We discover that a large fraction of non-coding somatic mutations in estrogen receptor (ER)-positive breast cancers are confined to ER binding sites. Notably, the highly mutated estrogen receptor binding sites are associated with more frequent chromatin loop contacts and the associated distal genes are expressed at higher level. To elucidate the functional significance of these non-coding mutations, we focus on two of the recurrently mutated estrogen receptor binding sites. Our bioinformatics and biochemical analysis suggest loss of DNA-protein interactions due to the recurrent mutations. Through CRISPR interference, we find that the recurrently mutated regulatory element at the LRRC3C-GSDMA locus impacts the expression of multiple distal genes. Using a CRISPR base editor, we show that the recurrent C→T conversion at the ZNF143 locus results in decreased TF binding, increased chromatin loop formation, and increased expression of multiple distal genes. This single point mutation mediates reduced response to estradiol-induced cell proliferation but increased resistance to tamoxifen-induced growth inhibition.

**Conclusions:**

Our data suggest that ER binding is associated with localized accumulation of somatic mutations, some of which affect chromatin architecture, distal gene expression, and cellular phenotypes in ER-positive breast cancer.

**Electronic supplementary material:**

The online version of this article (10.1186/s13059-018-1572-4) contains supplementary material, which is available to authorized users.

## Introduction

Somatic mutations are the driving force for cancer cell evolution [1]. Large-scale efforts, including The Cancer Genome Atlas (TCGA) [2] and International Cancer Genome Consortium (ICGC) [3], have mapped somatic mutations genome-wide in multiple cancer types. Beyond the protein-coding component of the genome, these whole-genome sequencing (WGS) efforts revealed that somatic mutation burden largely resides within non-coding genomic regions [4–8]. Since identification of the highly recurrent *TERT* promoter mutations, which occur in 50 of 70 (71%) melanomas examined at that time [9, 10], recurrent non-coding mutations have been discovered in promoters of *PLEKHS1*, *WDR74*, and *SDHD* in a pan-cancer analysis of 863 human tumors [5]. With more WGS data available for any given tumor type, more recurrent somatic mutations have been determined in the non-coding regions of specific cancers. For example, the promoters of protein-coding genes *PLEKHS1*, *WDR74*, and *TBC1D12* as well as long intergenic non-coding RNAs (lincRNA) *MALAT1* and *NEAT1* are recurrently mutated in breast cancer [4, 11].

Although technical advances in sequencing technologies and analytical pipelines empower us to better detect somatic mutations, our understanding of their origins and functional consequences are far from complete. Unlike the driver mutations inherited from the germ line, a variety of mutational processes may lead to distinct patterns of cancer type-specific somatic mutation accumulation during the lifetime of cancer patients [12, 13]. Causes of mutations such as mutagen exposures, aberrant DNA editing, and replication errors are known to uniformly affect the genome [14]. On the other hand, for cancers driven by external mutagens such as tobacco smoking in lung cancer and UV radiation in melanomas, differential chromatin accessibility and recruitment of nucleotide excision repair (NER) machineries have been proposed as major contributors for regional variation of mutation rate [15–17]. However, for most other cancers, the underlying mutational processes are not known. In this study, we examined whole-genome somatic mutations in 560 breast cancers in order to understand the biological processes and the regulatory impacts of recurrent non-coding mutations in breast cancer.

Breast cancer is the number one cause of cancer-related deaths in women [18, 19]. At the molecular level, it is mostly driven by aberrant hormonal activity of estrogens and estrogen receptor (ER). Estradiol (E2, 17β-estradiol), which is a natural hormone ligand for ERα, is essential for normal development and function of mammary tissue [20]. Paradoxically, a persistently elevated blood level of estrogen is causally linked to increased breast cancer incidence [21, 22]. Although how estrogen causes malignant mammary development is unclear, we hypothesized that there might be a mechanistic link between ER, the major transcription factor (TF) mediating estrogen response in breast cancer, and localized non-coding mutational load in the ER-positive breast cancer genome.

To test the hypothesis, we integrated whole-genome breast cancer sequencing data from 560 primary tumors (referred to as BRCA-EU) [4] with ChIP-seq identified ER binding events obtained from > 20 primary as well as metastatic ER-positive breast tumors (referred to as ER ChIP-seq) [23]. The integrative analysis shows a disproportionately large amount of somatic mutations at ER binding sites (ERBS). Importantly, we find that the highly mutated sites make more frequent chromatin loops and their target genes are expressed at higher levels. We also identified multiple uncharacterized recurrent (existing in more than one patient) non-coding mutations at ERBS. By utilizing the CRISPR interference and CRISPR base editing approaches, we interrogated the functional roles of two of these recurrent non-coding mutations in breast cancer cells. Bioinformatics, biochemical, and functional interference results at the chromatin as well as genetic levels suggest that these non-coding mutations alter expression of multiple distal genes through changes in non-ER TF binding and 3D DNA topology, and differentially modulate the cellular response to estradiol-induced cell proliferation and tamoxifen-induced growth inhibition.

## Results

To assess the relationship between ER binding activity and somatic mutation accumulation in breast cancer, we investigated whether there is increased mutational frequency at ERBS. We, therefore, acquired ER DNA binding profiles from *Ross-Innes* et al. (ER ChIP-seq) [23] for eight good-outcome ER^+^, progesterone receptor (PR)^+^, and HER2^−^, seven poor-outcome (ER^+^ PR^−^ HER2^−^ or ER^+^ PR^+^ HER2^+^) primary breast tumors, and three ER^+^ distal metastatic tumors from women with breast cancer. The original ER ChIP-seq study also included two breast cancer samples that were ER^−^ (ERα-negative), but expressed high transcript levels of ERβ as a control for ERα-specific binding events. By aggregating the ERBS identified by the two methods described in the original ER ChIP-seq study (MACS [24] and SWEMBL), we determined 253,908 ERBS in total for the 21 samples (including separate sections from the same tumor) [23]. To ensure the generalizability of this study, we used ERBS detected in at least two independent patients (*N* = 67,267), except for the particular analysis on ERBS shared by different numbers of patients. For the mutation data, we leveraged genome-wide somatic mutations identified through whole-genome sequencing of 560 ER^+^ HER2^−^ normal-matched breast tumors (BRCA-EU) [4]. Simple somatic mutations including 3,430,287 single base substitutions, 255,203 deletions, and 92,372 insertions of ≤ 200 bp were used in this study. Multiple base substitutions were not incorporated because of its limited number (*N* = 2680). With these two high-throughput genome-wide data sets from primary breast tumors (BRCA-EU and ER ChIP-seq), we aimed to decipher the in vivo mutational landscape underlying this lineage specifying TF-ER and identify the regulatory impact of non-coding somatic mutations associated with breast cancer.

**Fig. 1:**
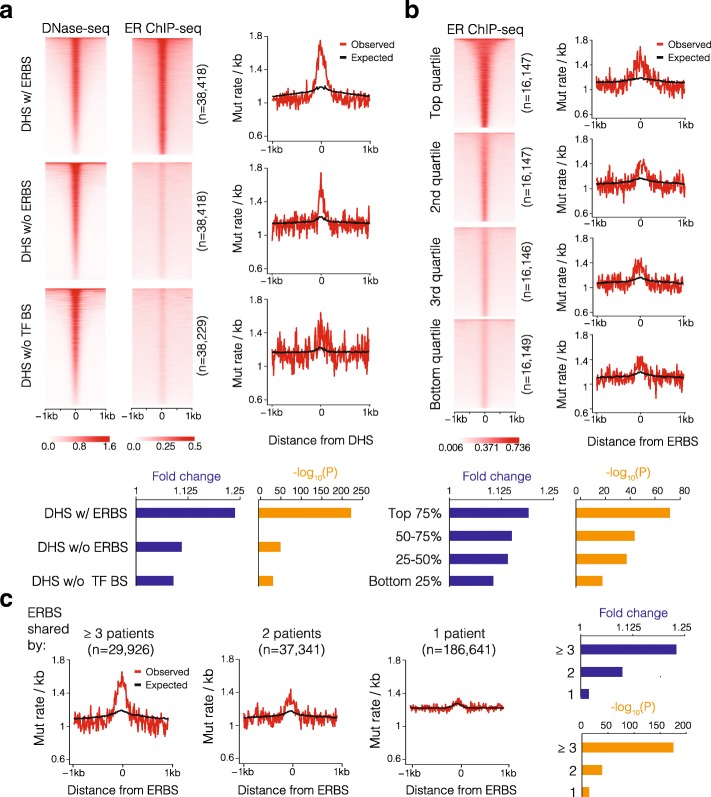
ER binding is associated with increased somatic mutation rates in breast cancer. Heatmaps show DNase I sequencing read intensity as a measure of DNase hypersensitivity in MCF-7 cells (ENCODE) and ER ChIP-seq read intensity in 21 ER^+^ breast cancer samples profiled by Ross-Innes et al. [23]. Observed somatic mutation rates (red line) for 560 ER^+^ breast cancer patients (ICGC BRCA-EU) [4] were calculated for sites with different ER binding and DNase hypersensitivity intensity. Expected mutation rates (black line) were calculated based on tri-nucleotide compositions of corresponding genomic sequences using previously established method [15]. Fold changes (blue bar) are comparing the observed mutation rates within 200 bp of ER binding or DHS peaks with the rates in flanking regions (> 200 bp and ≤ 1 kb); corresponding *P* values (orange bar) were obtained using chi-square test followed by Benjamini-Hochberg adjustment. **a** The observed and expected mutation rates were calculated for three sets of DHS sites with comparable intensity: the sites that overlapped with ER ChIP-seq peaks (DHS w/ ERBS), the sites that overlapped with other ENCODE identified TF but not ER binding sites (DHS w/o ERBS) and finally DHS with no TF binding sites (DHS w/o TF BS). **b** The observed and expected somatic mutation rates for four quartiles of ER binding sites with increasing ER binding intensity are shown. **c** The observed and expected somatic mutation rates at ERBS shared by more than 3 patients, 2 patients, and patient-specific are shown. Fold changes and *P* values are shown for each set of ERBS as described above

Since chromatin organization is a major contributor to mutation rates in the genome [25], we also included global chromatin accessibility data as measured by high-throughput sequencing of DNase I hypersensitive sites (DNase-seq) in MCF-7 breast cancer cells, which are ER-positive. To differentially investigate the mutation rates at ER-specific binding sites versus globally accessible DNase I hypersensitive sites (DHS), we divided all the identified DHS in terms of their overlap with the ER ChIP-seq peaks. We also selected an equal number of DHS that do not overlap with ER or other ENCODE-mapped TF binding sites. We then calculated the mutation rates at ± 1 kb around the center of the DHS sites. Notably, in these three sets of genomic regions with comparable DNase-seq signal intensity, we observed the highest rate of somatic mutations in DHS overlapping with ERBS (fold change [FC] = 1.24, chi-square test *P* = 1.81 × 10^−233^; Fig. 1a). This elevated mutation rate could not be explained by the expected mutation rate of the corresponding tri-nucleotide sequence context [13]. We also observed substantial mutation burden at DHS without ER binding but bound by other ENCODE-mapped TFs (FC = 1.11, chi-square test *P* = 2.16 × 10^−55^; Fig. 1a). This enrichment was significantly lower than the DHS sites with ER binding (two-sided ks test *P* = 1.82 × 10^−10^), suggesting that, to a lesser extent, additional TFs may also contribute to the localized mutation burden. The DHS without ER and any ENCODE-mapped TF binding had the lowest mutation burden (FC = 1.09, chi-square test *P* = 4.68 × 10^−36^; Fig. 1a). These results suggest that TF binding in general, particularly ER binding, is strongly associated with increased somatic mutation burden in breast cancer beyond the effect of open chromatin states.

Next, we investigated if ER binding intensity is differentially associated with somatic mutation rates. We separated ERBS into quartiles based on the binding intensity in ER ChIP-seq. We found that there are substantially more somatic mutations at sites with stronger ER binding (FC = 1.19, chi-square test *P* = 9.26 × 10^−73^) and a positive correlation (Pearson correlation coefficient = 0.9) between ER binding intensity and the somatic mutation rate (Fig. 1b). In line with this, the genomic regions that are constitutively bound by ER (from ER ChIP-Seq data) across different patients have significantly higher mutation rates, suggesting that more commonly used ERBS contain more somatic mutations (FC = 1.23, chi-square test *P* = 5.60 × 10^−176^, Fig. 1c). In contrast, the patient-specific ERBS have near background-level mutation rates (Fig. 1c). Notably, since commonly used ERBS tend to be the high-intensity ones [23], the above noted observation remains significant (Wald test *P* = 1.11 × 10^−8^) even after controlling for ERBS binding intensity using a negative binomial generalized linear regression model (Additional file 1: Figure S1). Thus, both ER binding intensity and frequency among independent patients are associated with increased somatic mutation burden (Additional files 2 and 3). The ERBS located in promoter, intronic, or intergenic regions contain comparable levels of mutations (Additional file 1: Figure S2). Interestingly, when we analyzed the insertions and deletions (≤ 200 bp) at ERBS, we observed negative association between ER binding activity and the rate of insertions/deletions, suggesting a potentially protective effect (Additional file 1: Figure S3). When we separated all the single nucleotide mutations into the six possible nucleotide changes, we observed significant enrichment for C>G and C>T mutations at ERBS, which is indicative of an APOBEC mutational signature, consistent with the genome-wide trend reported by Nik-Zainal et al. [4], Morganella et al. [26], and Periyasamy et al. [27] (Additional file 1: Figure S4).

**Fig. 2:**
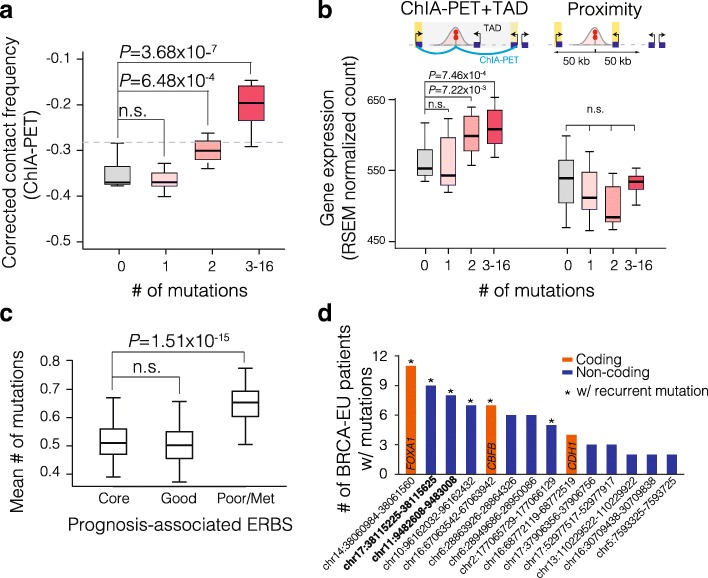
Frequently mutated ERBS are associated with more chromatin loops and higher gene expression. Boxplots presented in this figure illustrate the lower quartile (Q1) and higher quartile (Q3) as the box, median as the line inside the box, and 1.5 × interquartile range (IQR = Q3 - Q1) as the whiskers. **a** Boxplot depicts corrected long-range chromatin contact frequency from Pol2 ChIA-PET data in MCF-7 cells, for ERBS with different numbers of somatic mutations in BRCA-EU. The contact frequency was corrected using a negative binomial linear regression model to remove the effect of ER binding intensity (Additional file 1: Figure S5). Gray dash line indicates the average corrected contact frequency for all ERBS. **b** Boxplot represents expression levels of genes that are topologically associated (within the same TAD and associated with ERBS via ChIA-PET loop) or linearly associated (50 kb distance) with ERBS. ERBS were grouped according to the number of mutations within 200 bps of its summit (same as in panel **a**). **c** Mean number of somatic mutations is plotted for ERBS that are associated with good outcome, poor outcome/metastasis and shared by at least 75% of breast cancer patients (core ERBS) [23]. The average mutation number was calculated based on random sampling of 100 ERBS from each group for 50 times. *P* values are calculated using two-sided Student’s *t* test. **d** Bar plot shows the number of BRCA-EU patients carrying mutations at the ERBS, which contained the most number of somatic mutations within 200 bps of the summit (except for FOXA1, somatic mutations ~ 100 bps beyond the 200-bp limit were included due to its recurrence) across all the patients. Asterisk indicates if there are recurrent mutations (existing in at least two BRCA-EU patients). Gene symbols for the ERBS within coding regions are shown inside the bars. The two ERBS that are characterized in this study are in bold font

These data support our hypothesis that the binding activity of ER is associated with increased somatic mutation rates. Next, we studied whether the mutated ERBS are differentially associated with transcription and chromatin organization. We therefore integrated ER binding and mutation data with gene expression and 3D chromatin organization assayed by RNA pol II Chromatin Interaction Analysis by Paired-End Tag Sequencing (ChIA-PET) [28] and Hi-C mediated topologically associating domains (TADs) in MCF-7 cells [29]. Notably, the highly mutated ERBS make more frequent chromatin interactions (corrected for ER binding intensity; Fig. 2a, Additional file 1: Figure S5). More importantly, we observed that genes topologically associated with highly mutated ERBS (within the same TAD and forms a ChIA-PET loop) are expressed at significantly higher levels (two-sided *t* test *P* = 7.46 × 10^−4^ for ERBS with 3–16 mutations, Fig. 2b). Critically, when the same analysis is performed based on 2D-linear proximity, the genes proximal to the same sites (within 50 kb) are not expressed significantly higher (Fig. 2b). These results indicate that highly mutated ERBS are involved in regulation of multiple target genes [30] through long-range chromatin interactions and support the concept of “transcription factories” [31].

We next investigated whether ERBS that are associated with good-outcome or poor-outcome and metastatic breast tumors have differential mutation burden. Using the pre-defined ERBS from Ross-Innes et al. (ER ChIP-seq) [23], we found that the poor outcome/metastasis-specific ERBS (poor/met ERBS) were significantly more mutated than both the good outcome-specific ERBS (good ERBS; two-sided *t* test *P* < 2.2 × 10^−16^) and the constitutively bound common ERBS (core ERBS; two-sided *t* test *P* = 2.30 × 10^−3^; Fig. 2c). A negative binomial linear regression model confirmed the higher mutation rate at poor/met ERBS (Wald test *P* = 2.57 × 10^−3^) after correcting for ER binding intensity and number of chromatin interactions at ERBS (Additional file 1: Figure S6a). This model suggests that the number of mutations at ERBS is independently associated with patients’ clinical outcome. Interestingly, utilizing the sequencing reads from the ER ChIP-seq data, we identified multiple potential somatic mutations at the ERBS (Additional file 1: Figure S6b, Additional file 2). Notably, consistent with our observations using the BRCA-EU WGS data, we observed a higher percentage of mutations at poor ERBS in samples with poor outcome/metastasis (Additional file 1: Figure S6b).

The vast majority of ERBS (98% in this study; Additional file 1: Figure S2) are within non-coding regulatory DNA elements [23]. Thus, a large fraction of the somatic mutations that we identified at ERBS do not alter coding sequences. Among the most highly mutated ERBS, only three overlap with coding regions, which correspond to three driver genes *(FOXA1*, *CBFB*, and *CDH1*) reported previously (Fig. 2d) [4]. The next critical challenge is to characterize the regulatory impact of non-coding mutations. We focused our efforts on recurrent non-coding mutations at ERBS that are detected in at least two independent patients. We reasoned that such mutations might have higher regulatory impact due to their selective advantage in tumor evolution. The top recurrent non-coding somatic mutations were functionally characterized (Fig. 2d).

**Fig. 3:**
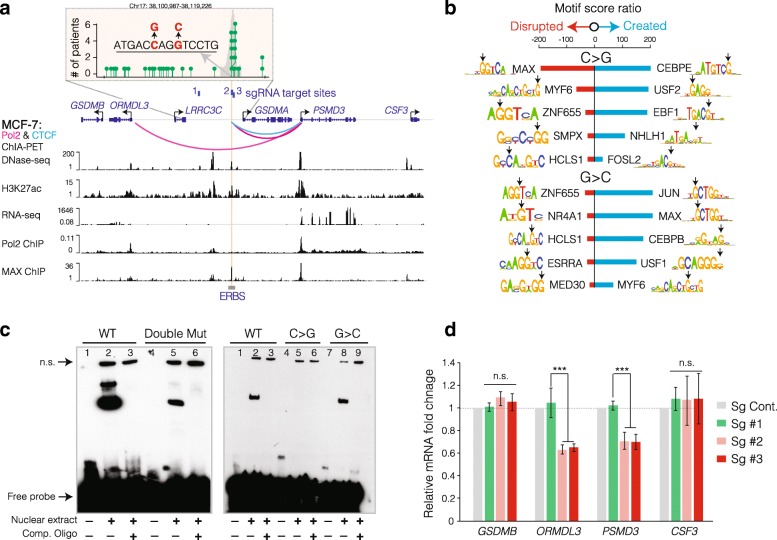
A recurrent intergenic somatic mutation disrupts TF binding and decreases expression of distal genes. **a** Genomic region of two recurrent somatic mutations and their neighboring genes is shown. Inset shows the number of BRCA-EU patients with mutations in the intergenic locus between the *LRRC3C* and *GSDMA* genes. Nucleotide changes for the two recurrent mutations and the relative position of the ER peak (gray shadow) are shown. Relevant tracks (ENCODE) and positions of the sgRNAs used in panel **d** are also displayed. **b** Motif scores were calculated with and without each mutation using the PWMEnrich package [57], which performs DNA motif enrichment analysis against databases such as MotifDb. Motif score ratios were displayed as blue and red bars representing higher motif scores with and without the mutation, respectively. Downward black arrows indicate the mutation position within each motif. **c** EMSA results demonstrate protein binding affinity for WT and mutant oligonucleotides (oligos) with either double or single mutations. The three lanes for each case are biotin-labeled oligos only, biotin-labeled oligos plus nuclear extract, and biotin-labeled oligos plus nuclear extract and competitor probes from left to right. Competitor probes are unlabeled oligos to examine DNA-protein binding specificity. Non-specific interactions are labeled as “n.s.”. **d** Neighboring gene expression levels were assessed by qRT-PCR in MCF-7 cells with CRISPR-dCas9-based interference of control and the mutation sites. All the *P* values were calculated with two-sided Student’s *t* test. ***P < 0.001. Error bars represent standard deviations from six biological replicates

In an intergenic locus between the *LRRC3C* and *GSDMA* genes, the two recurrent mutations are only two base pair away from each other (Fig. 3a). We detected the C→G conversion in five BRCA-EU patients whereas the G→C mutations in six patients; of these patients with the mutant alleles, two patients carry both mutations. To study the potential regulatory function of these mutations, we performed bioinformatics analysis to see the probabilities of which TF motifs are changed the most in the presence of these mutations. The analysis results suggest that the probabilities of MYC-associated factor X (MAX) motif is decreased the most by the C→G mutation whereas the G→C mutation is more likely to create novel TF motifs (Fig. 3b). Interestingly, the in vitro electrophoretic mobility shift assay (EMSA), which measures the biochemical affinity of proteins in cellular extract to a given oligonucleotide sequence, shows that the C→G mutation alone was responsible for most of the diminished protein binding affinity, while the G→C mutation had minimal effect, indicating that the C→G recurrent mutation is likely disrupting TF binding activity (Fig. 3c). Notably, ENCODE ChIP-seq data shows that MAX is among the TFs that strongly bind to this locus (Fig. 3a) [28]. Our computational and biochemical results led to the hypothesis that the recurrent C→G mutation disrupts TF-DNA interaction at this site. To further study the potential gene targets and the functional role of the mutation in an in vitro setting, we utilized the CRISPR interference assay by targeting catalytically inactive dCas9 to the recurrent mutation site in MCF-7 cells, which are wild type for the mutations. Previously, through ChIP-seq analysis, we showed that dCas9 strongly associates with DNA and occupies an ~ 150 bp genomic region [32]. We designed four separate sgRNAs: a non-genome targeting control, another control that targets a 4-kb distal non-regulatory genomic site, and two separate sgRNAs with slightly overlapping guiding sequences that target the mutation site. Upon targeting dCas9 with these sgRNAs, we measured transcriptional alterations in all genes within certain spatial proximity as well as genes that are topologically associated with the mutation site. It is worth noting that we only observed significant reduction in the mRNA levels of the *ORMDL3* and *PSMD3* genes, which are topologically associated with the mutation site based on both Pol II and CTCF ChIA-PET data (Fig. 3a, d). The two immediate proximal genes instead, *LRRC3C* and *GSDMA*, neither interact with the mutation site nor expressed at detectable levels in either the MCF-7 breast cancer cell line (Fig. 3a) or primary breast tumors (TCGA) [33]. Variants of ORMDL3 (ORMDL Sphingolipid Biosynthesis Regulator 3) were expressed in different breast cancer cell lines [34]. Although the functional relevance of ORMDL3 to breast cancer has not been reported, it has been shown to be differentially expressed in ER^+^ tumors [34]. *PSMD3* (proteasome 26S subunit, non-ATPase 3), encoding a member of the proteasome subunit, may participate in numerous cellular processes, including cell cycle progression, apoptosis, or DNA damage repair. Silencing of *PSMD3* has been shown to have an additive inhibition of cell viability as well as induced apoptosis in HER2^+^ breast cancer cells [35]. However, its functional role in HER2^−^ cells is not characterized.

**Fig. 4:**
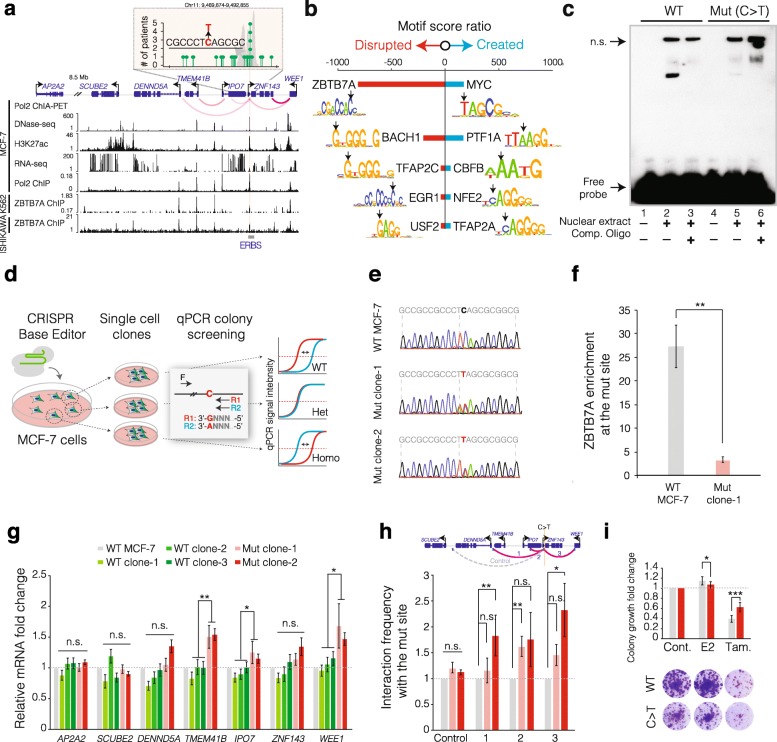
A recurrent non-coding somatic mutation at the *ZNF143* locus affects TF binding, 3D chromatin architecture and expression of multiple distal genes. **a** Genomic region of the recurrent mutation at the *ZNF143* promoter and the neighboring genes is shown. Inset shows the number of BRCA-EU patients with mutations around the *ZNF143* promoter. The sequence flanking the C to T mutation and the relative position of the ER peak (gray shadow) to the mutations are shown. Relevant ENCODE sequencing tracks are also displayed. **b** Motif score ratios were calculated between genomic sequences with and without the mutation. Blue bars indicate higher motif scores with the mutation, thus motif created; red bars represent higher motif scores without the mutation, thus motif disrupted. Downward black arrows indicate the mutation position within each motif. **c** EMSA results demonstrate protein binding affinity for WT and mutant (with the C>T mutation; Mut) oligonucleotides. The three lanes for each case are biotin-labeled oligos only, biotin-labeled oligos plus nuclear extract, and biotin-labeled oligos plus nuclear extract and competitor probes from left to right. Competitor probes are unlabeled oligos to examine DNA-protein binding specificity. Non-specific interactions are labeled as “n.s.”. **d** Schematic representation of the CRISPR base editor approach to introduce the C to T mutation into MCF-7 cells. qPCR was utilized to screen genomes of more than 400 single cell colonies to detect the specific mutation. **e** Sanger sequencing results show the genomic sequences at and around the mutation site in WT and two mutant (Mut) MCF-7 clones. **f** ChIP-qPCR analysis shows ZBTB7A enrichment at the mutation site in MCF-7 WT cells and a mutant clone. Error bars represent standard errors of four independent data points (biological replicates). The *P* value was calculated using one-sided Student’s *t* test. **g** qRT-PCR results show relative mRNA levels of genes that are topologically or spatially associated with the mutant site in WT and mutant MCF-7 clones. Error bars represent standard deviations from 11 biological replicates. **h** Bar graphs show contact frequency between the mutated site and the four other proximal sites in WT and mutant MCF-7 cells as measured by the Chromatin Conformation Capture (3C) assay. Interacting sites from the MCF-7 Pol2 ChIA-PET data are colored in magenta. Hypothetical interaction with the control site is indicated with a gray dash line. The blue boxes at the end of the interaction curves indicate the primer positions used in the 3C assay. Error bars represent standard deviations (2 biological replicates). **i** Crystal violet colony formation assay measures the relative size and viability of colonies for WT and mutant MCF-7 cells in response to control, estradiol (E2) and tamoxifen (Tam.) treatment. Images and corresponding quantifications are shown. Error bars represent standard deviations from 12 biological replicates. All the *P* values were calculated with two-sided Student’s *t* test unless indicated otherwise. ***P < 0.001, **P < 0.01, *P < 0.05

The second highly mutated ERBS we investigated is within the promoter of *ZNF143*, with the recurrent mutation (C→T) present in five independent patients (Fig. 4a). Our motif analysis indicated that this mutation significantly disrupts the binding of ZBTB7A (Fig. 4b). In line with this, the EMSA results suggested significant reduction of protein binding affinity to the mutant oligos (Fig. 4c). ZBTB7A ChIP-seq data is not available in breast cancer cell lines. However, although TF binding is context and cell line-specific, the publicly available data for K562 leukemia and Ishikawa endometrial cancer cells show strong ZBTB7A binding to this site (Fig. 4a) [28]. To more comprehensively characterize the regulatory function of this mutation, we used the CRISPR base editor technology [36] to engineer the exact mutation in breast cancer cells. The CRISPR base editor (BE3) is exploiting cytidine deaminase activity of the APOBEC enzyme, which is fused to a nickase Cas9 and results in a direct C→T conversion at the target site without DNA double-strand breaks. We recently used the CRISPR base editor to introduce early STOP codons as a safer approach to induce gene silencing [37]. To create minimal experimental artifacts, we transiently transfected the BE3 complex with an sgRNA targeting the mutation site to introduce the C→T mutation in MCF-7 cells, which contain the wild-type allele (Fig. 4d). Since we aimed to identify clones with a single point mutation, we devised a qPCR screening approach where a C→T conversion results in ~ 2 ΔCt difference in qPCR signals (Additional file 1: Figure S7). Using this strategy, we screened the genomic DNA isolated from ~ 400 single cell expanded colonies and identified multiple clones with one copy of the mutant T allele at the desired position (Fig. 4d, e). Then, we examined whether ZBTB7A binding is altered as predicted by the computational motif analysis and EMSA results. Critically, our ChIP-qPCR analysis shows that the ZBTB7A enrichment is reduced approximately eightfold in the mutant cells compared to WT MCF-7 cells (one-sided *t* test *P* = 0.006; Fig. 4f). Analysis in an independent mutant clone showed comparable reduction in ZBTB7A enrichment at the target site (Additional file 1: Figure S8). ZBTB7A binding is 125 bps upstream of ER binding summit in ER ChIP-seq. How its decreased binding affects ER binding is unknown. ZBTB7A is a member of the POK (POZ/BTB and Krüppel) transcription repressors [38, 39]. We therefore checked with qRT-PCR to see if the expression of proximal and topologically associated genes is altered. Notably, the expression of three genes (*TMEM41B*, *IPO7* and *WEE1*) was significantly increased (two-sided *t* test *P* = 8.87 × 10^−3^ for *TMEM41B*, *P* = 0.03 for *IPO7*, *P* = 0.04 for *WEE1*) in two independent mutant clones compared to clonal wild-type MCF-7 cells (Fig. 4g). Since these genes are topologically associated with the mutation site (Fig. 4a), we next investigated whether the mutation not only disrupts TF binding, but also alters the 3D DNA topology of the locus. As expected, we observed a higher frequency of loop formation between the mutation site and the target genes in the mutant MCF-7 clones as assessed by the 3C (Chromosome Conformation Capture) approach (Fig. 4h) [40].

Initially, we anticipated this recurrent mutation to confer a proliferative advantage on MCF-7 cells. Contrary to our expectation, we did not observe increased cell proliferation in the mutant clones. However, we noticed that these mutant cells are less responsive to estradiol-mediated cell proliferation. This result led to the hypothesis that the mutation is contributing to hormone independent growth, which is a characteristic of late-stage breast cancer. We therefore investigated to see if the mutation renders cells partially resistant to tamoxifen. Notably, we observed significant resistance to tamoxifen-induced growth inhibition in the mutant cells (paired two-sided *t* test *P* = 7.5 × 10^−4^, Fig. 4i). It is notable that the clinical patients’ survival data (METABRIC) [41] shows that higher expression of *TMEM41B*, *IPO7*, and *WEE1* are significantly associated with poor outcome (Additional file 1: Figure S9). The *TMEM41B* encodes the transmembrane protein 41B, and its functional role in breast cancer progression is not known. The *IPO7* gene encodes Importin 7 protein, which regulates the import of specific ribosomal proteins for ribosomal assembly. Its expression is stimulated by Myc and suppressed by p53 [42], rendering it a possible pro-survival gene through ribosomal biogenesis. Importin-7 also regulates nuclear transport of steroid hormone receptors, including the androgen receptor [43]. The tyrosine kinase encoded by *WEE1* is a crucial component of the G2-M cell cycle checkpoint that prevents entry into mitosis in response to cellular DNA damage [44]. Normal cells repair damaged DNA during G1 arrest; however, cancer cells, often with a deficient G1-S checkpoint, depend on a functional G2-M checkpoint for DNA repair [44]. And indeed WEE1 is found to be expressed at high levels in various cancer types including breast cancer [45] and has been identified as one of the molecules in the tamoxifen resistance pathway [46]. Moreover, WEE1 inhibition has already been reported to synergistically inhibit breast cancer growth in combination with cisplatin in xenograft models [47].

## Discussion

Cancer arises due to aberrant regulation of multiple genes and signaling pathways [14, 48]. Although genetic alterations in selected oncogenes and tumor suppressors may initiate the process of cellular transformation, additional mutations contribute to the evolution of cancer cells. WGS efforts in large cohorts of patients have improved our ability to identify candidate driver mutations in the coding and noncoding genomic regions. However, defining the pathways associated with DNA mutagenesis and understanding the impact of cancer-associated mutations in non-coding genomic regions are yet to be complete.

Our results presented here suggest that ER binding is associated with increased mutation burden at the binding site. The molecular mechanism that mediates accumulation of somatic mutations at and around ERBS is yet to be identified. Estrogen, the hormone that activates ER, was shown to potentiate DNA damage nearly three decades ago [49]. However, why estrogen treatment results in increased DNA damage and whether this is due to increased ER binding activity is not fully understood. We postulate three potential mechanisms to explain the relatively higher rates of somatic mutations at ERBS. Firstly, somatic mutations may originate stochastically or induced by the by-products of estrogen metabolism, such as quinones [50]. These mutations will likely be evenly distributed across the genome. However, the mutations at ERBS may be repaired at a reduced rate due to the physical presence of ER, which may block DNA repair machinery. This may result in relatively higher mutational frequency at and around ERBS [15]. Secondly, some of these stochastically emerged or metabolite induced mutations may be selected during tissue development or tumor evolution due to their functional impacts. These acquired somatic mutations may alter ER or other TF binding, affect gene expression and drive cancer progression. Thirdly, the ER binding activity or ER induced transcriptional process may directly induce somatic mutations at ERBS by causing strand breaks or exposing the local DNA to deaminases such as APOBE3B [27]. To this end, a plausible molecular mechanism is transcription coupled R-loop formation at and around ERBS [51]. R-loop formation at distal enhancers and actively transcribed genes conflicts with DNA replication machinery, and is a known process that aggravates DNA damage and activates various DNA repair mechanisms [52]. It is possible that all these three mechanisms contribute to the ERBS mutations. Our results presented here and currently available genomic data does not allow us to differentiate among the three mechanisms. The recurrent somatic mutations that we report here could be examples of the second mechanism where a mutation is selected during tumor evolution. On the other hand, the observation that the mutated ER binding sites are topologically associated with highly expressed genes is supportive of the third mechanism. Regardless of the original molecular mechanism that causes these mutations, our functional characterization efforts show that some of the ERBS mutations may alter gene expression by affecting the binding activities of TFs as shown for the C-to-T mutation at the *ZNF143* locus.

Critically, our findings also suggest that somatic mutations at ERBS may accumulate in normal/pre-neoplastic breast tissue as well. In line with this, we observed significant more somatic mutations at ERBS when we used blood instead of tumor adjacent breast tissue as “normal” in the mutation calling process (chi-square test *P* = 7.20 × 10^−32^ [*N* = 60] vs *P* = 2.18 × 10^−3^ [*N* = 10], Additional file 1: Figure S10a), suggesting that somatic mutations at ERBS exist in pre-neoplastic breast cells (Additional file 1: Figure S10b). Since the control tissue type for 87.5% of the BRCA-EU patients was unknown, this hypothesis needs to be further investigated using additional WGS samples, ideally matched blood and breast tissues from normal individuals.

Although identifying whole-genome level cancer mutations is now feasible, understanding the functional significance of a vast number of coding and non-coding mutations and identifying their gene targets remain a major challenge. To this end, we employed multiple complementary novel approaches to characterize the two recurrently mutated non-coding genomic regions. We demonstrate that the CRISPR interference and base editor approaches can be utilized to characterize functional significance of a single point mutation. Unlike biochemical EMSA or plasmid-based luciferase assay, such tools allow interrogating endogenous chromatin loci for their downstream effects, which is more informative as shown by the *ZNF143* locus. Specifically, a recent study by Rheinbay et al. [11] discovered this very same mutation through customized exome-capture sequencing of an independent cohort of 360 breast cancer patients. Their subsequent EMSA and luciferase promoter analysis indicated the functional impact of this mutation on *ZNF143* gene expression, but our genetic base editing data suggest that although expression of *ZNF143* is slightly altered, the major regulatory impact of the mutation is observed at the topologically associated distal genes. These findings further highlight the power of utilizing CRISPR editing approaches in combination with ENCODE-derived topological data in characterizing the functional roles and identifying the potential targets of non-coding mutations in the genome. To this end, the CRISPR base editor tools, which are being significantly expanded to edit not only C•G to T•A transitions but also A•T base pairs to G•C [53], will be immensely useful to interrogate the regulatory impact of various non-coding mutations in cancer as well as other diseases.

It is notable that the two recurrent mutations that we identified and functionally interrogated here are implicated in the expression of multiple distal genes. Such findings demonstrate that it is important to study the regulatory effect of non-coding mutations beyond the most proximal promoter. In addition to the recurrent mutations, it remains to be studied whether non-recurrent mutations also contribute to differential survival or proliferation in cancer cell evolution. Since the rate of recurrence is simply determined by the number of patients carrying the mutant allele in the BRCA-EU cohort, it is likely that many other non-coding mutations may have “driver” functionality in cancer evolution.

## Methods

### Data accession and preprocessing

#### Whole-genome sequencing data (BRCA-EU)

Whole-genome somatic mutations of 560 ER^+^ and HER2^−^ breast cancer patients (BRCA-EU) in International Cancer Genome Consortium (ICGC) were obtained from ICGC Data Portal (https://dcc.icgc.org) [4, 26]. Simple somatic mutations including 3,430,287 single base substitutions, 255,203 deletions, and 92,372 insertions of ≤ 200 bp detected in the original study were used here. Multiple base substitutions were not analyzed because of its limited number (*n* = 2680).

#### ER ChIP-seq data

ER binding sites (ERBS) from 18 independent ER^+^ breast cancer patients were obtained from Gene Expression Omnibus (GEO; GSE32222) [23]. Three patient samples have two sections sequenced separately in the original study to detect tumor heterogeneity [23]. The genomic coordinates of ER binding sites were lifted from hg18 to hg19 to be consistent with the mutation coordinate (https://genome.ucsc.edu/cgi-bin/hgLiftOver). Both MACS [24] and SWEMBL (https://www.ebi.ac.uk/~swilder/SWEMBL/) identified ER binding events from the original study [23] were used in this study to derive ERBS shared in different numbers of patients. BEDTools multiIntersect [54] was used to carry out this operation. For all analysis except for Fig. 1c, ERBS shared by at least two patients were used. One thousand one hundred ninety-two genomic regions that had significantly stronger ER binding in the patients with poor outcome or metastasis compared to the good outcome patients, 599 ERBS with stronger ER binding in the good outcome patients when compared to the poor/met patients, and a core set of 484 ERBS that were identified in at least 75% of all the tumors, but not in either of the ER- tumors were obtained from the original study (www.carroll-lab.org.uk/data) [23]. Alignment files (bam files) for each sample were downloaded for potential somatic mutation identification from GEO.

#### DNase-seq data

The genomic coordinates of DNase I hypersensitivity sites (DHS) in MCF-7 cells were obtained from ENCODE (GSE29692) [28]. DHS with more than 50% overlap between the two replicates of samples either with (GSM1024784 and GSM1024783) or without (GSM1024764 and GSM1024767) estradiol treatment were used in our analysis. Since DHS in MCF-7 cells with and without hormone treatment were highly correlated with each other (minimum pearson correlation coefficient of 0.93), and both sets of DHS provided the same analysis results, we only showed the results using DHS with estradiol treatment in Fig. 1a.

#### RNA-seq data

Whole transcriptome data of 1093 breast cancer patients identified by The Cancer Genome Atlas (TCGA) were obtained using the TCGAbiolinks R package [33, 55]. RSEM normalized results of gene expression were used, which divide the raw counts by the 75th percentile of read counts for each sample and then multiply by 1000. For each gene, its median expression value among the 1093 samples was calculated for downstream analysis.

#### ChIA-PET, Hi-C, and other ChIP-seq data

Pol2 ChIA-PET and Hi-C data in MCF-7 cells were obtained from ENCODE (GSE39495) [28] and GEO (GSE66733), respectively. Relevant ChIP-seq data sets for H3K27ac, Pol2, MAX, and ZBTB7A in MCF-7 or other cell lines were located on the ENCODE website (https://www.encodeproject.org) and visualized through the UCSC genome browser (https://genome.ucsc.edu).

### Mutation rates estimation

#### Observed rate

For each analysis, the included ER binding sites or DHS were extended 1 kb on both sides from their peak summit positions (5 kb for insertions and deletions) to compare the mutation rate in the intervals we are interested in to their flanking regions. We excluded any regions overlapping coding sequences and UCSC Browser blacklisted regions, often misaligned to sites in the reference assembly (Duke and DAC), and with low unique mappability of sequencing reads. For analysis centered on ER binding sites, regions that overlap other TF binding sites within flanking regions were also excluded. All TF binding sites from ENCODE were obtained from the UCSC genome browser (https://genome.ucsc.edu). After the filtering step, mutation data were mapped to ER binding or DHS intervals, and mutation rate at the nucleotide resolution was computed and plotted.

#### Expected rate

For each analyzed interval sets, we calculated the probabilities of occurrence of all possible 96 tri-nucleotide changes (similar to computing mutation signatures) [13]. And then the mean expected mutation rate, after 1000 times random sampling, based on sequence context (tri-nucleotide compositions) at each nucleotide position was plotted against the observed rate for comparison. For each time of random sampling, the number of different 96 tri-nucleotide changes was kept the same. Expected mutation rate was not calculated for insertions and deletions, due to unavailability of robust methods to predict their occurrence based on sequence context.

### Mutation enrichment analysis

The fold change and *P* value between mutation rates within 200 bp of ER binding or DHS peak summits and flanking regions (> 200 bp and ≤ 1000 bp) were modeled using a chi-square distribution. The obtained *P* values were corrected for multiple testing using the Benjamini-Hochberg procedure [56].

### Negative binomial linear regression models

All the regression models were built using the glm.nb function in R. The final fitted model was determined by performing ANOVA test for models with different independent variables included. We also compared the final negative binomial model with a corresponding Poisson model. *P* values for the coefficients of included independent variables were calculated using Wald test. To use any model for prediction, 1000 data points for each independent variable were independently simulated. Then the model was used to predict values for the response variable. To remove effects of any independent variable on the response variable, residuals function in R was applied to obtain the corrected values of the response variable.

### Gene expression analysis

#### ChIA-PET based

For ERBS containing different numbers of mutations within 200 bps of its summit, we grouped them into ERBS with 0, 1, 2, and ≥ 3 mutations. Then, we randomly sampled 500 regions from each ERBS group and repeated the sampling for 10 times. Choosing 500 random sites was limited by the number of ERBS with at least 3 mutations. Next, the random sampled ERBS set was intersected with ChIA-PET data in MCF-7 cells to obtain their interacting sites. Genes overlap the interacting sites and within the same TADs as the ERBS based on Hi-C MCF-7 insulation boundaries (40 kb resolution) were selected for further analysis. To make sure an equal number of genes were included for each ERBS group, we randomly sampled 200 genes for 100 times and computed the mean expression levels of the genes sampled each time. The distributions of gene expression levels were compared across ERBS groups with different numbers of mutations. *P* values were calculated using two-sided *t* test.

#### Proximity based

For the same ERBS randomly sampled in the above ChIA-PET based analysis, genes intersected with regions 50 kb or 100 kb flanking the ERBS summits were obtained. Same as above, 200 genes were randomly sampled for 100 times to compute mean expression distributions for different ERBS groups, which avoids bias when comparing with the ChIA-PET based analysis results. *P* values were calculated using two-sided *t* test.

### Somatic mutation detection from ER Chip-Seq

Since corresponding control tissues are not available for the ER ChIP-seq data, we used mutation sites that are identified from the 560 WGS BRCA-EU samples [4, 26] and are within 200 bps of ERBS summits as potential somatic mutation sites. Bam files for the 9 ER ChIP-seq samples with good outcome, and the 12 samples with poor or metastasis outcome were merged for mutation discovery [23]. Then, we used bam-readcount (https://github.com/genome/bam-readcount) to get the counts of different alleles covering the potential mutation sites in the two merged bam files. To increase the credibility of somatic mutations identified from the ChIP-seq data, only the sites covered with at least 10 reads and encompassing both the reference and alternative alleles in BRCA-EU were selected as potential somatic mutations. In the end, the percentage of outcome-associated ERBS that contain potential somatic mutations was calculated for samples with corresponding outcomes.

### Motif analysis

Nucleotide sequences with and without the mutations for the *LRRC3C* and *GSDMA* intergenic and *ZNF143* loci were processed with the PWMEnrich R package, to detect motifs significantly enriched [57]. Motifs with scores ≥ 100, roughly equivalent to *P* value ≤ 10^−3^ in either the reference or mutant sequence were considered to be confidently identified. Motif score ratios between mutant and reference sequences were calculated for the reliably identified motifs. Motifs with large absolute values of score ratios were presented in Figs. 3b and 4b.

### Computational and statistical tools

BEDTools utilities [54] were used to carry out operations such as extensions or overlaps in the various analyses of genomic features. Ngs.plot was used to generate heatmaps for DHS and ER binding intensities [58]. All the statistical tests were performed in the R (version 3.4.1) and python 3.5.2 environment. Customized bash, R and python scripts were used to perform all the other analysis.

### Experimental assays

#### Electrophoretic mobility shift assay (EMSA)

EMSAs were performed using a ThermoFisher Scientific LightShift Chemiluminescent EMSA kit following the manufacturer’s instructions. MCF-7 cell nuclear extracts were prepared using NE-PER Nuclear and Cytoplasmic Extraction Reagents (ThermoFisher Scientific) according to the manufacturer’s protocol. 20 fM biotin-labeled probes were used for each EMSA reaction. Increasing amounts of unlabelled WT or mutant competitor oligonucleotides were used to analyze specificity of mobility shifts. Competitor probe concentration was 8 pM. Reactions were incubated for 20 min at room temperature, size-separated on a 6% native polyacrylamide gel, and transferred to a Biodyne B Nylon membrane (ThermoFisher Scientific). Free or protein-bound biotin-labeled probes were detected using streptavidin-horseradish peroxidase conjugates and chemiluminescent substrate according to the manufacturer’s instructions. Membranes were placed in a film cassette and exposed to X-ray for 1–2 min. Probe sequences for the *LRRC3C* and *GSDMA* intergenic region are WT: CCGCATGACCAGGTCCTGCTTC, double mutations: CCGCATGACGAGCTCCTGCTTC, C>G single mutation: CCGCATGACGAGGTCCTGCTTC, and G>C single mutation: CCGCATGACCAGCTCCTGCTTC; for the *ZNF143* promoter region are WT: CCGCCGCCCTCAGCGCGGCGG and mutant: CCGCCGCCCTTAGCGCGGCGG.

#### CRISPR/dCas9 interference

Promega FuGene 6 (cat. no. E2691) was used for transient transfection according to the manufacturer’s protocol. 70% confluent MCF-7 cells were used for each transfection. The same molar ratio of plasmids were used for dCas9-target and control sgRNAs. We used 6 μg dCas9 and 2 μg sgRNA plasmids per 10 cm plate. After 36 h, puromycin (2 μg/mL) was added to select transfected cells. SgRNA sequences used are as follows: Sg Cont.: GGAGCGCACCATCTTCTTCA, Sg #1: GCGAGGCAGGAGGATTGCTTG, Sg #2: GCAGCACTCACCGCATGACC, and Sg #3: GGAAGCAGGACCTGGTCATG.

#### Real-time qRT-PCR

Total RNA from MCF-7 cells was extracted by using the QIAGEN RNeasy Mini Kit according to the manufacturer’s protocol. RNA was reverse transcribed using the High-Capacity RNA-to-cDNA kit (Applied Biosystems), and cDNA was amplified using the QuantiFast SYBR Green PCR Kit. CT values of target genes were normalized to *GAPDH*.

#### CRISPR base editor

MCF-7 wild-type cells were cultured at 37 °C with 5% CO_2_ in the DMEM media containing 10% fetal bovine serum (FBS) and 1% penicillin–streptomycin. For transient transfection, dCas9-APOBEC3 and the target sgRNA were transfected by Promega FuGene 6 (cat. no. E2691) into 50–70% confluent MCF-7 cells [37]. After transfection for 2–3 days, the cells were diluted and then seeded in 15 cm dishes to grow colonies. Single colonies were picked up, grown in 96-well plates, and then transferred to 24-well plates for expansion. The sgRNA sequence used for CRISPR base editor is GGCCCTCAGCGCGGCGGCGC.

#### qPCR colony screening

For each colony, genomic DNA was isolated according to the protocol [37] and qPCR was performed using the primers that cover the point mutation site of the *ZNF143* gene. The qPCR primer sequences are: F: GGTGGTCGGACGAAGGAATT; R1: GGCCCGCGCCGCCGCGCTG; R2: GGCCCGCGCCGCCGCGCTA. For positive colonies, their PCR products were submitted for Sanger sequencing at Eton Biosciences.

#### ChIP-qPCR

MCF-7 WT and mutant cells from two 15-cm plates were subjected to previously published ChIP protocol [59, 60]. Briefly, cells were cross-linked with 1% formaldehyde for 10 min and neutralized with final 0.125 M glycine for 5 min at 37 °C. Pellets were lysed in SDS lysis buffer and incubated for 20 min on ice. The chromatin was sonicated using Branson digital sonifier for 9 min at 40% amplitude with 0.7 s “on” and 1.3 s “off” pulse cycles. Fragmented chromatin was diluted with ChIP-dilution buffer (0.01% SDS, 1.1% Triton X-100, 1.2 mM EDTA and 16.7 mM Tris-HCl, pH 8.1) and incubated with 1.5 μg ZBTB7A antibody (abcam # 106592) overnight at 4 °C. After overnight incubation, 30 μl mixture of protein A-G magnetic beads (Dynabeads, Life Technologies) were added to lysates and rotated for 2 h at 4 °C. Next, beads were washed well on the magnetic field with each of these buffers two times: low-salt immune complex wash buffer (0.1% SDS, 1% Triton X-100, 2 mM EDTA, 20 mM Tris-HCl [pH 8.1] and 150 mM NaCl); LiCl wash buffer (0.25 M LiCl, 1% NP40, 1% deoxycholate, 1 mM EDTA and 10 mM Tris-HCl [pH 8.1]); and TE (10 mM Tris-HCl and 1 mM EDTA [pH 8.0]). The chromatin was recovered from the beads by 30 min incubation with elution buffer (0.2% SDS and 0.1 M NaHCO3 supplemented with fresh 5 mM DTT) at 65 °C. After reverse cross-linking, proteinase K and RNase digestion, DNA was extracted with ethanol precipitation method and quantified via Qubit Fluorometer. Purified DNA from immunoprecipitation was used to analyze the fold enrichment of ZBTB7A at the mutation site of *ZNF143*. Two primer pairs close to the mutation site were used to analyze the ZBTB7A enrichment. The two primer pairs are: F1: GGTGGTCGGACGAAGGAATT, R1: GCCAGGCGGAGAATAATGCA; F2: GGCCTTGCCGATTTTATGGG, R2: AAAAAGCTCCGCCGCCTAG. Fold-enrichment ratios were calculated by the ΔΔCt method by using IP DNA and WCE (whole cell extract) DNA as a control input. A primer pair for a negative control genomic region was used to calculate fold enrichment. The negative control primers are as follows: F: AAAAATCAGTTTGTGTGTTTGTGG, R: CCTAGGCAAC AGTGACACCTATTT.

#### Chromosome conformation capture (3C) assay

3C experiment was performed as stated in Hagege et al. [61]. Briefly, 10 million MCF-7 cells were collected and crosslinked with 1% formaldehyde inside 10% FCS/PBS solution for 10 min at room temperature. Crosslinking was quenched with ice-cold 0.125 M glycine (final concentration) for 5 min. Sequentially, cell and nuclear membrane lysis reactions were performed with appropriate buffers. Nla*III* (NEB R0125) restriction enzyme was used for overnight digestion of the crosslinked genomic DNA. Ligation was carried out by using T4 Ligase at 16 °C for 4 h followed by Proteinase K (300 μg total) and RNase treatment (200 μg total). DNAs were precipitated by using phenol-chloroform extraction method. qPCR was performed by using primer pairs targeting the mutated site and one of the interacting sites. Normalized crosslinking frequency was calculated by using Ct value difference between target and genomic control primer pairs. Primer sequences used in this assay are as follows: for the mutated site: AGCTTCCATTGGGCTGTCAT; for the control site: GTCAATCTCCAGCCTGGATTCATCC; for the interacting site 1: GAGACTCCTTTAGGGAGGGC; for the interacting site 2: GGGATCATTTGAAGTCAGGAGTTC; for the interacting site 3: TAACTAGGAGTAGGCCTAAGGG; for the genomic control site F: GGCATTGTTGATTCACGGGT and R: CAACGGGCAGAATGTAGCTC.

#### Crystal violet assay

Wild-type and mutant MCF-7 cells were plated at a density of 1,000 cells per well in 12-well plates. The next day estradiol (E2) and tamoxifen (2 μM) were added to the growth medium. Fresh media was added after 6–7 days. After ~ 14 days, the well would be washed twice with PBS, then stained for 30 min with crystal violet solution (0.4% crystal violet, 10% formaldehyde, 80% methanol). After staining, the crystal violet solution was removed, and then the stained cells were washed once with PBS and 3+ times with water. The plate was inverted overnight and covered to dry before imaging.

## Additional files

Additional file 1: **Figure S1.** ERBS shared by more patients contain more mutations after controlling for ER binding intensity. **Figure S2.** Comparable levels of somatic mutation burden are observed at ERBS overlapping promoter, intronic, and intergenic regions. **Figure S3.** ERBS are protected from somatic insertions and deletions, and the protective effect is correlated with ER binding intensity. **Figure S4.** Somatic mutations enriched at ERBS show the APOBEC mutational signature. **Figure S5.** ERBS with more mutations make more frequent chromatin interactions independent of ER binding intensity. **Figure S6.** ERBS associated with poor/met outcome contain more somatic mutations. **Figure S7.** Amplification plots for WT and mutant MCF-7 clones based on the qPCR colony screening strategy depicted in Figure 4d. **Figure S8.** Somatic mutation reduces ZBTB7A binding. ChIP-qPCR analysis shows ZBTB7A enrichment at the mutation site in MCF-7 WT cells and a mutant clone. **Figure S9.** High expression of TMEM41B, IPO7 and WEE1 is associated with poor survival for breast cancer patients. **Figure S10.** Somatic mutation burden at ERBS is higher when blood instead of adjacent to tumor breast tissue is used as “normal” in the mutation calling process. (PDF 2338 kb)

Additional file 2: **Table S1.** Variant annotation for good outcome-associated ERBS in ER ChIP-seq samples with good outcome. **Table S2.** Variant annotation for poor outcome/metastasis-associated ERBS in ER ChIP-seq samples with poor outcome/metastasis. These data are associated with Additional file 1: Figure S6b. (XLSX 66 kb)

Additional file 3: Review history. (DOCX 58 kb)
