# Supplementary material for: Recurrent mutations at estrogen receptor binding sites alter chromatin topology and distal gene expression in breast cancer
Source: Genome Biol. 2018 Nov 7;19:190. doi: 10.1186/s13059-018-1572-4 (PMC6223090; doi:10.1186/s13059-018-1572-4)
Supplement: Supplementary file 1 — Figure S1. ERBS shared by more patients contain more mutations after controlling for ER binding intensity. Figure S2. Comparable levels of somatic mutation burden are observed at ERBS overlapping promoter, intronic, and intergenic regions. Figure S3. ERBS are protected from somatic insertions and deletions, and the protective effect is correlated with ER binding intensity. Figure S4. Somatic mutations enriched at ERBS show the APOBEC mutational signature. Figure S5. ERBS with more mutations make more frequent chromatin interactions independent of ER binding intensity. Figure S6. ERBS associated with poor/met outcome contain more somatic mutations. Figure S7. Amplification plots for WT and mutant MCF-7 clones based on the qPCR colony screening strategy depicted in Figure 4d. Figure S8. Somatic mutation reduces ZBTB7A binding. ChIP-qPCR analysis shows ZBTB7A enrichment at the mutation site in MCF-7 WT cells and a mutant clone. Figure S9. High expression of TMEM41B, IPO7 and WEE1 is associated with poor survival for breast cancer patients. Figure S10. Somatic mutation burden at ERBS is higher when blood instead of adjacent to tumor breast tissue is used as “normal” in the mutation calling process. (PDF 2338 kb) [file 13059_2018_1572_MOESM1_ESM.pdf]

Figure S1

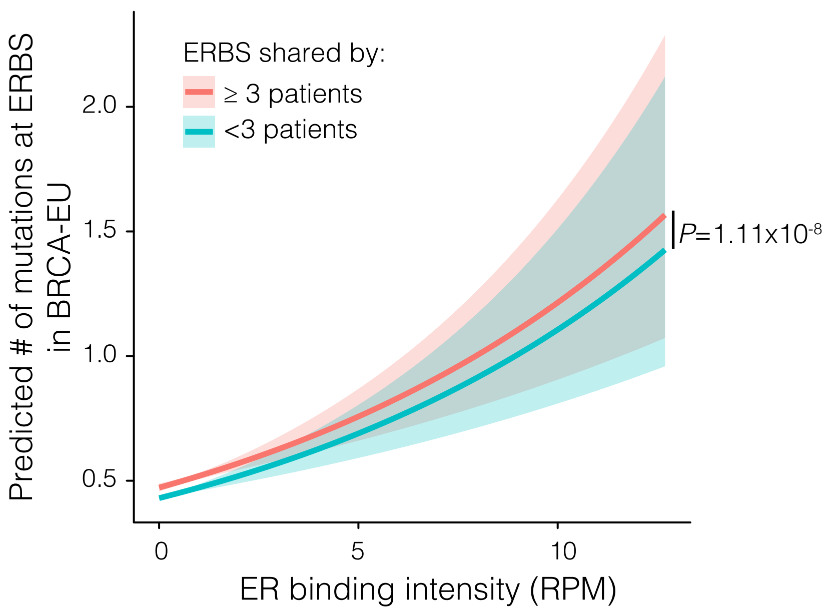

Figure S1: ERBS shared by more patients contain more mutations after controlling for ER binding intensity. A negative binomial linear regression model was built to predict the number of mutations within 200 bps of ERBS in BRCA-EU. ER binding intensity and the number of patients sharing the ERBS were used as predictors. Shades represent the 95% confidence interval (CI) of prediction lines with the same color. The P value was calculated using Wald test. RPM: reads per million mapped reads.

Figure S2

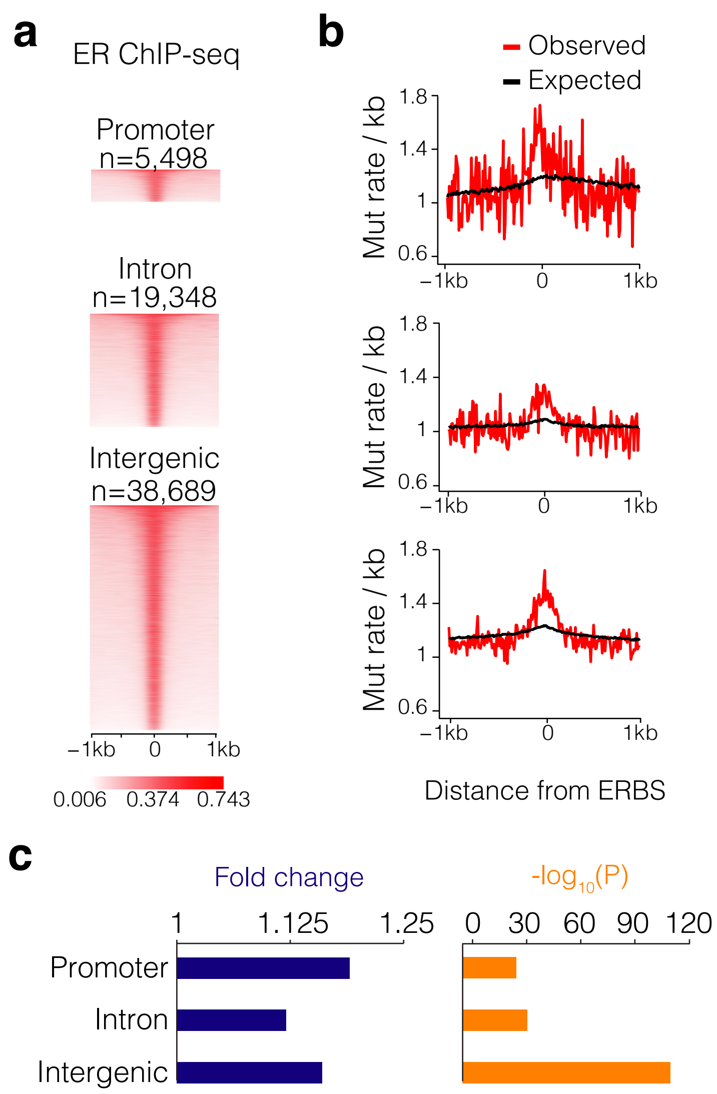

Figure S2: Comparable levels of somatic mutation burden are observed at ERBS overlapping promoter, intronic, and intergenic regions. (a) Heatmaps show ER binding intensity in the 21 ER ChIP-seq patients for ERBS overlapping promoter, intronic, and intergenic regions, respectively. (b) Observed somatic mutation rates (red line) for the 560 BRCA-EU patients were calculated for the three sets of ERBS as shown in panel a. Expected mutation rates (black line) were calculated based on tri-nucleotide compositions of corresponding genomic sequences using previously established method[16]. (c) Fold changes (blue bar) are comparing the observed mutation rate within 200 bp of ER binding peaks with the rate in flanking regions (>200 bp & ≤ 1 kb); corresponding P values (orange bar) were obtained using chi-square test followed by Benjamini-Hochberg adjustment.

Figure S3

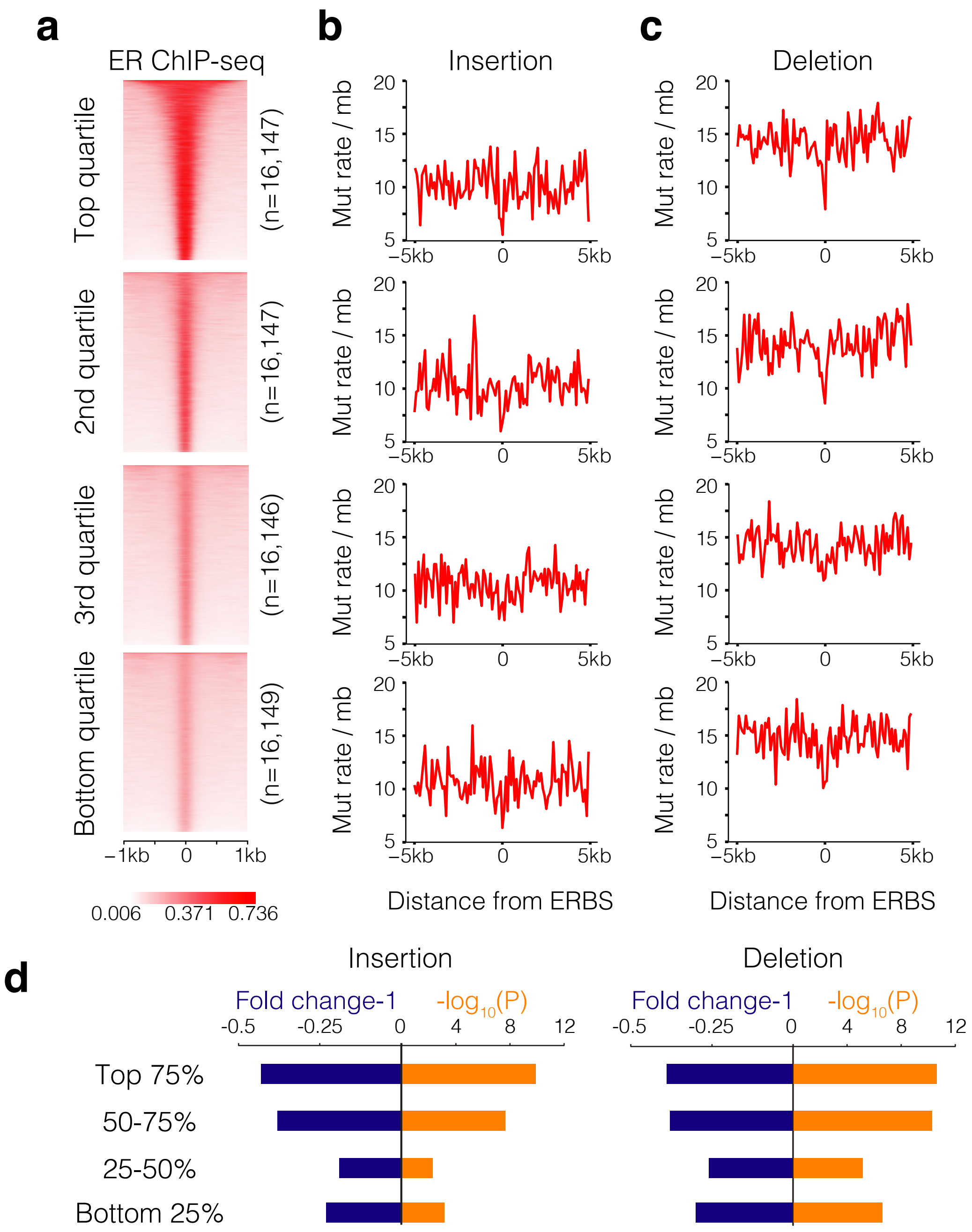

Figure S3: ERBS are protected from somatic insertions and deletions, and the protective effect is correlated with ER binding intensity. (a) Heatmaps show ER binding intensity quartiles in the 21 ER ChIP-seq patients. Insertion (b) and deletion (c) rates (red line) for the 560 BRCA-EU patients were calculated for the four quartiles of ERBS. (d) Fold changes (blue bar) are comparing the observed mutation rate within 200 bp of ER binding peaks and the rate in flanking regions (>200 bp & ≤ 1 kb), thus negative Fold change – 1 values show the protective effect of ER binding on insertions and deletions; P values (orange bar) were obtained using chi-square test followed by Benjamini-Hochberg adjustment.

Figure S4

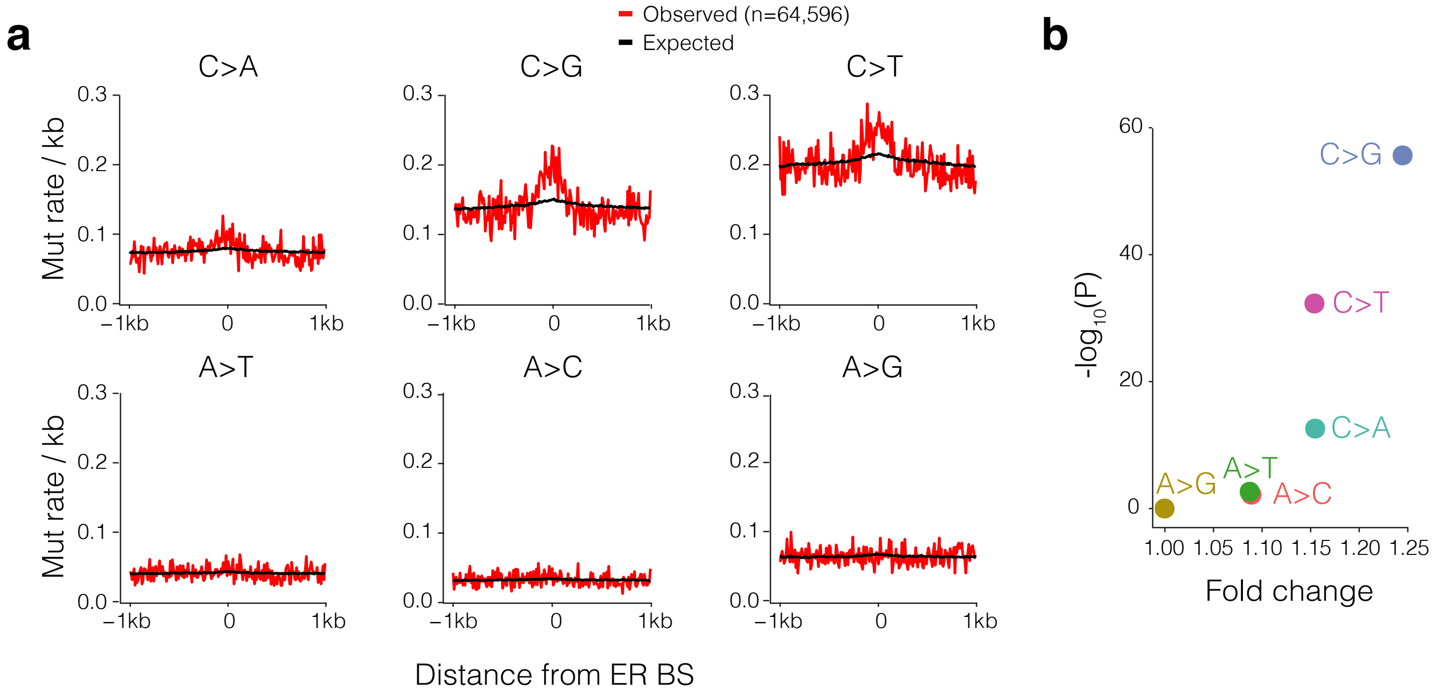

Figure S4: Somatic mutations enriched at ERBS show the APOBEC mutational signature. (a) Observed somatic mutation rates (red line) for the 560 BRCA-EU patients at all the ERBS detected in ER ChIP-seq were calculated for the six different nucleotide changes separately. Expected mutation rates (black line) were calculated based on tri-nucleotide compositions of corresponding genomic sequences using previously established method[16]. (b) Fold changes on the x axis represent the observed mutation rate within 200 bp of ER binding peaks and the rate in flanking regions (>200 bp & ≤ 1 kb); P values on the y axis were obtained using chi-square test followed by Benjamini -Hochberg adjustment. C>G and C>T mutations are most significantly enriched at ERBS.

Figure S5

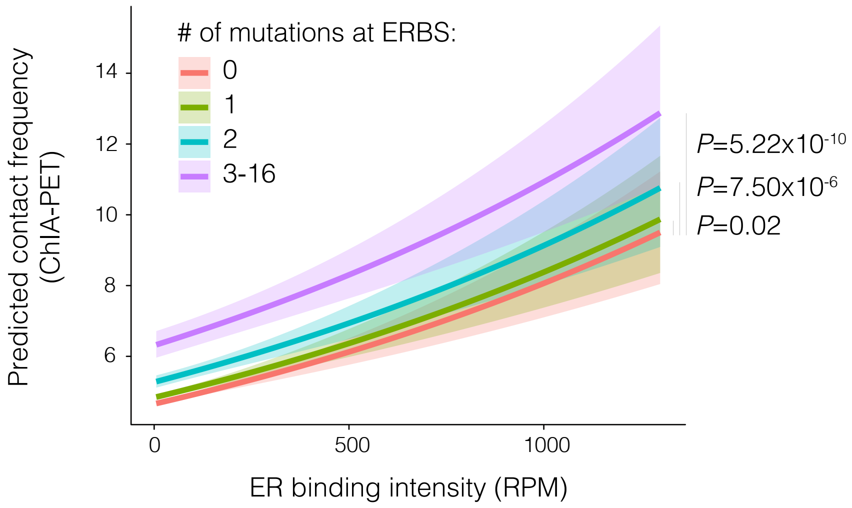

Figure S5: ERBS with more mutations make more frequent chromatin interactions independent of ER binding intensity. ChIA-PET contact frequency was predicted using the negative binomial linear regression model built upon the BRCA-EU data. One thousand ER binding intensity values were simulated between the observed minimum and maximum values from the ER ChIP-seq data. These values together with the ERBS mutation groups predicted the number of chromatin interactions for ERBS. Shades represent the 95% confidence interval (CI) of prediction lines with the same color. P values were calculated using Wald test. RPM: reads per million mapped reads.

Figure S6

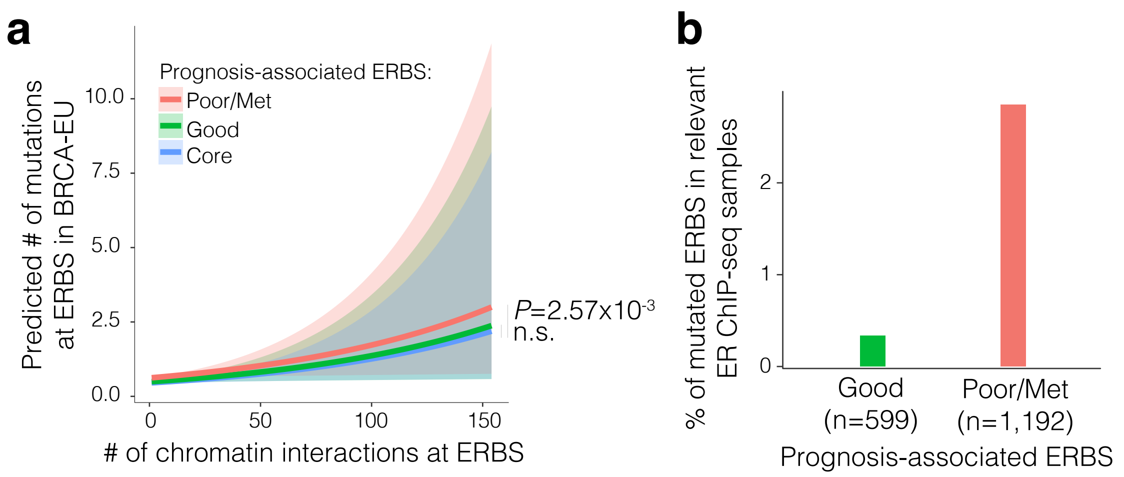

Figure S6: ERBS associated with poor/met outcome contain more somatic mutations. (a) Number of mutations per ERBS (within 200 bps of its summit) was predicted using a negative binomial linear regression model, which includes ER binding intensity based on ER ChIP-seq, number of chromatin interactions based on MCF-7 Pol2 ChIA-PET, and clinical outcome associated with the ERBS (good, poor/met or core). Core ERBS were defined as sharing by at least 75% of the ER ChIP-seq patients[24]. Since in the final fitted model ER binding intensity is not a significant predictor (Wald test P = 0.48), it is not included in the plot. (b) Potential somatic mutations at ERBS associated with good or poor/met outcomes were identified in the ER ChIP-seq samples. An ERBS was considered as mutated if there was at least one potential somatic mutation. The percentage of mutated ERBS that are associated with good or poor/met outcomes was plotted for the ER ChIP-seq samples with good or poor/met outcomes, respectively. The associated raw data are in **Additional File 2**.

Figure S7

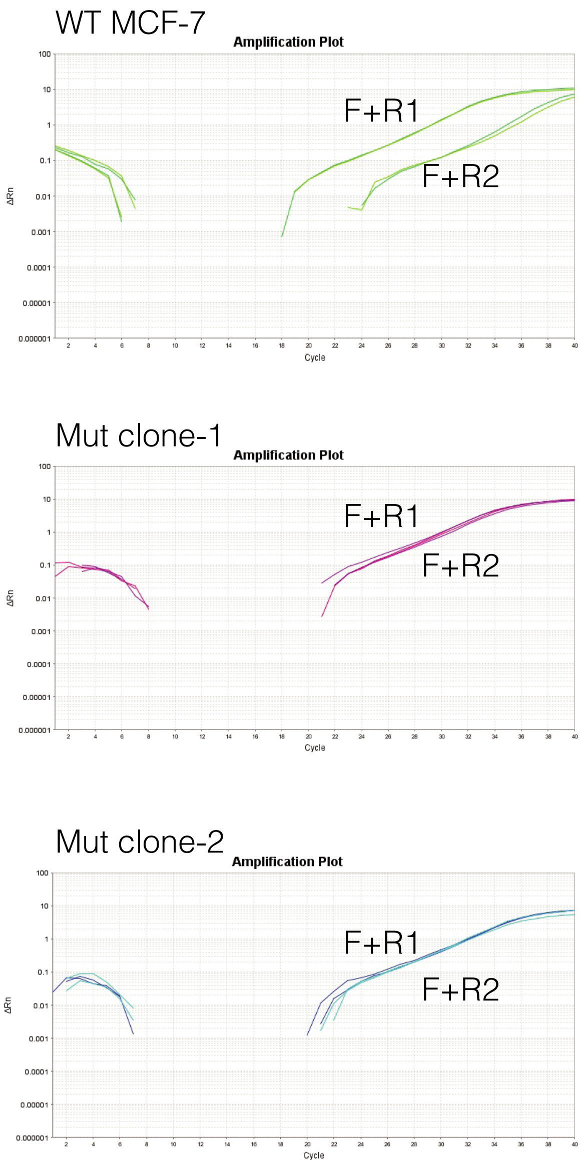

Figure S7: Amplification plots for WT and mutant MCF-7 clones based on the qPCR colony screening strategy depicted in Figure 4d.

Figure S8

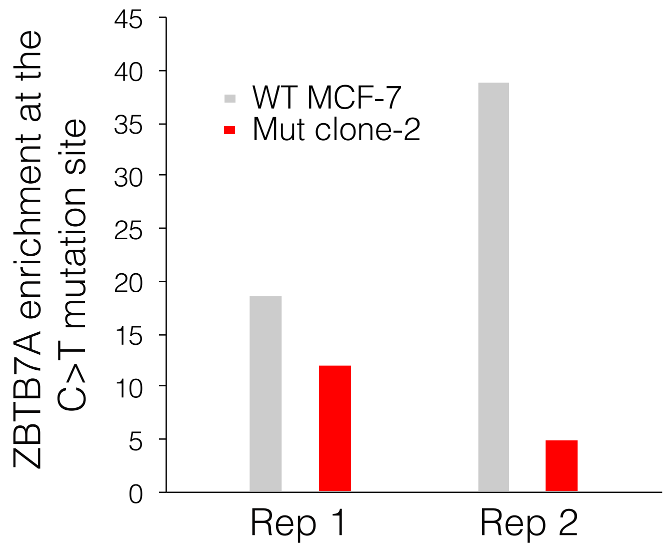

Figure S8: Somatic mutation reduces ZBTB7A binding. ChIP-qPCR analysis shows ZBTB7A enrichment at the mutation site in MCF-7 WT cells and a mutant clone. Results from two biological replicates are shown.

Figure S9

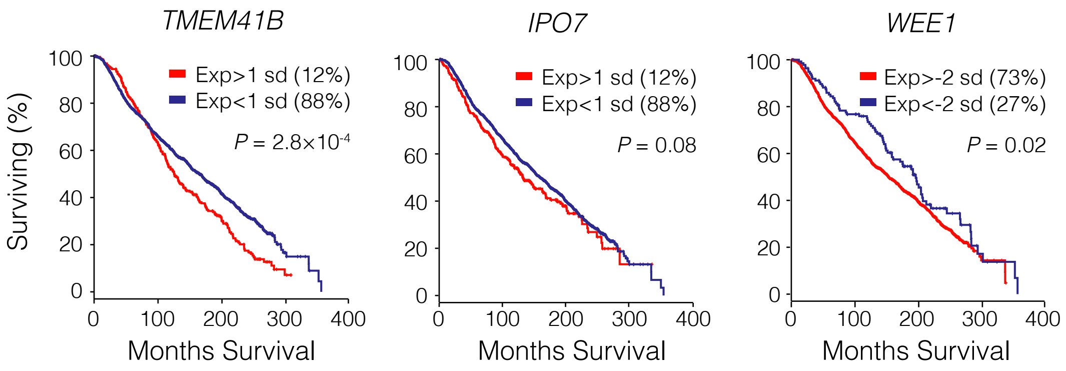

Figure S9: High expression of TMEM41B, IPO7 and WEE1 is associated with poor survival for breast cancer patients. Kaplan-Meier plots of overall survival are shown for patients from the METABRIC study with different expression levels of TMEM41B, IPO7 and WEE1. Log-rank P values are provided.

Figure S10

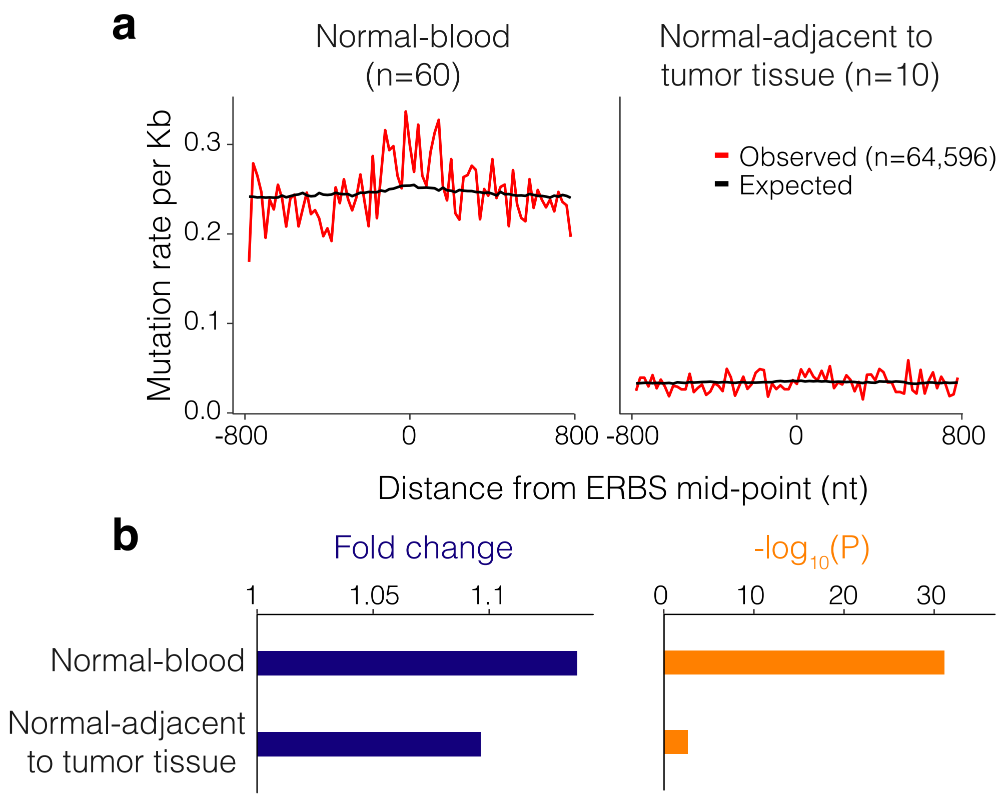

Figure S10: Somatic mutation burden at ERBS is higher when blood instead of adjacent to tumor breast tissue is used as "normal" in the mutation calling process. (a) Observed somatic mutation rates (red line) for the 60 BRCA-EU patients using blood as "normal" and the 10 patients using adjacent to tumor breast tissue as "normal" were calculated for all the ERBS detected in ER ChIP-seq. Expected mutation rates (black line) were calculated based on tri-nucleotide compositions of corresponding genomic sequences using previously established method[16]. (b) Fold changes (blue bar) are comparing the observed mutation rate within 200 bp of ER binding peaks with the rate in flanking regions (>200 bp & ≤ 1 kb); corresponding P values (orange bar) were obtained using chi-square test followed by Benjamini-Hochberg adjustment.
