# Supplementary material for: Recurrent mutations at estrogen receptor binding sites alter chromatin topology and distal gene expression in breast cancer
Source: Genome Biol. 2018 Nov 7;19:190. doi: 10.1186/s13059-018-1572-4 (PMC6223090; doi:10.1186/s13059-018-1572-4)
Supplement: Supplementary file 3 — Review history. (DOCX 58 kb) [file 13059_2018_1572_MOESM3_ESM.docx]

Review History

**First round of review**

**Reviewer 1**

**Are the methods appropriate to the aims of the study, are they well described, and are necessary controls included?**
Yes, methods are appropriate to support most conclusions.

**Are the conclusions adequately supported by the data shown?**

no, not all.

multiple variables that are being reported in the study are actually linked, while this is presented as independent factors. Mutations are found at ER sites, shared by multiple tumors, strongest sites, and involved in ChIA-PET loops. However, the strongest sites are always the ones shared by multiple tumors, and there is an intrinsic bias in the ChIA-PET data towards the strongest sites. Consequently, all these variables are linked and consequently not interpretable as separate entities. This could also explain the differential mutational frequency at the good and poor outcome ER sites, and may thus represent an artefact.

It is stated in figure 4H that tamoxifen-induced cell death is blocked, but tamoxifen does not induce cell death at physiologically relevant concentrations, and the data shown in Figure 4H do not depict cell death, but colony growth instead; this is something completely different.

In Figures 3 and 4, computational analyses are performed to show disrupted or created transcription factor binding motifs when a specific mutation is introduced. These data should be validated experimentally, by performing ChIP-QPCR analyses in the CRISPR models they've generated.

for other issues, please see comments for the authors.

**Are sufficient details provided to allow replication and comparison with related analyses that may have been performed?**

Yes

**Does the work represent a significant advance over previously published studies? Please explain.**

yes

**Is the paper of broad interest to others in the field, or of outstanding interest to a broad audience of biologists?**

Yes: the study assesses somatic mutations at Estrogen Receptor sites, and the potential implications thereof on downstream gene expression. This is relevant and of broad interest.

Comments to authors:

In this manuscript, Yang and colleagues studied overlap of somatic mutations in breast cancer with Estrogen Receptor binding events. An enrichment of somatic mutations at ER sites was observed, more specifically in those strong regions shared by multiple tumors, involved in 3D genome architecture. Using CRISPR, the authors altered specific ER sites, impacting 3D chromatin interactions (3C) and downstream gene expression. In general, I think this is an interesting paper, making intelligent use of many publicly available resources. Nonetheless, some results are in my opinion over-interpreted, and cause-effect relationships between ER binding and somatic mutations cannot be concluded given the data that is presented.

major comments:

1. It is stated in the manuscript, that specific enrichment of APOBEC-type mutations are observed at the ER sites. This is in line with work reported by Periyasamy et al., 2015 Cell Reports. This work should be acknowledged.

2. Multiple variables that are being reported in the study are actually linked, while this is presented as independent factors. Mutations are found at ER sites that shared by multiple tumors (Figure 1C), strongest sites (Figure 1B), and involved in ChIA-PET loops (Figure 2A). However, the strongest sites are always the ones shared by multiple tumors, and there is an intrinsic bias in the ChIA-PET data towards the strongest sites. Consequently, all these variables are linked and consequently not interpretable as separate entities. This could also very well explain the differential mutational frequency at the good and poor outcome ER sites (Figure 2C), and may thus represent an artefact.

3. The ChIA-PET data as described in Figure 2 should be confirmed with Hi-C data. Also, the analyses in Figure 2B should be reperformed, taking TADs into consideration.

4. It is stated in figure 4H that tamoxifen-induced cell death is blocked, but tamoxifen does not induce cell death at physiologically relevant concentrations, and the data shown in Figure 4H do not depict cell death, but colony growth instead; this is something completely different.

5. The introduction states that breast cancer is the most-common cancer related death. This is incorrect (lung cancer is; Ferlay et al., 2015), and this should be corrected.

6. The hypothesis that forms the basis for the work, is that Estrogen Receptor chromatin interactions could play a causal role inducing somatic mutations. However, the current study does not address this hypothesis, as cause and effect are not separated with the current experimental design. With the current data, the authors did not convince this reviewer that ER binding would represent a mechanism of somatic mutation induction; it could simply represent an evolutionary pressure on stochastic events that ultimately alter the biological role of the main driver of the disease.

7. the analyses in Figure 4H indicate a specific somatic mutation to 'decrease tamoxifen-induced cell death (even though I don't agree with this, see point 4). As the patient samples used for somatic mutation analyses and the tumor samples used for ChIP-seq analyses were from treatment -naive patients, these tumors have not encountered tamoxifen yet. Does this represent a disconnect?

8. Are the 'good outcome' and 'poor outcome' Estrogen Receptor sites used in this study actually mutated in the samples that were used in the ChIP-seq analyses? As raw sequencing data for these ChIP-seq samples is available from the Ross-Innes paper, the authors should determine whether mutations are in fact associated with the differential ER binding in these very same samples. This is a critical point.

9. Is outcome data available for the patients for which WGS data was generated? Are somatic mutations at the observed sites also associated with outcome in these patients?

10. In Figures 3 and 4, computational analyses are performed to show disrupted or created transcription factor binding motifs when a specific mutation is introduced. These data should be validated experimentally, by performing ChIP-QPCR analyses in the CRISPR models the authors have generated. This is a critical point.

11. As enhancer-activity is highly variable between tissue types, I feel it is not appropriate to use ChIP-seq data of cell lines from other tumor types to support the claims in the paper (Figure 4A).

12. the last part of the results section describing Figure 4 is quite elaborate, and the authors should consider moving parts to the discussion section.

**Reviewer 2**

**Are the methods appropriate to the aims of the study, are they well described, and are necessary controls included?**
The methods used in this study are appropriate for the aims, and represent a logical approach to addressing a very complex question about non-coding recurrent mutations in breast cancer genomes and their impact on the onset and progression of disease. The methods are reasonably well described, although there are several very confusing points in the text and in the figure legends that will need to be addressed in a revised version of the manuscript. In terms of controls, the only control missing from the analysis is one that provides information about where constitutive ER binding is occurring in normal breast cells. All the data utilized in these analyses is derived from breast cancer samples or breast cancer cell lines. Much interpretation about the mutations at ER binding sites is interpreted in the absence of a comparator normal data set for ER occupancy from ChIPseq. This proves ultimately to be a challenge for the resulting conclusions made by the authors, as detailed below.

Much of the confusion about methods stems from the lack of clear definition in the text about the different samples and data sets being used in the study. It would be advantageous to refer to the different data sets by a common identifier that would permit the reader to understand which samples are being referred to, rather than wondering which samples were, in fact, being discussed. Here is an example from page 11: "We detected the C to G conversion in five tumors whereas the G to C mutations in six patients; of these patients with the mutant alleles, two carry both mutations." In a single sentence, you have referred to tumors and patients and the meaning of anything from this sentence is subject to a lot of interpretation.

Finally, the methods description of ChIA-PET based gene expression analysis (pg 31) needs to be re-written so it is understandable. At present, there are grammatical problems that make the first few sentences even more difficult to interpret.

**Are the conclusions adequately supported by the data shown?**

In general, the conclusions arrived at from the data analysis and functional experiments were supported by the data shown, with one exception. My concern is that your interpretation of the coincidence of ER binding sites and higher than average mutation rates is not interpreted correctly from your data. Your conclusion is that ER binding is the mechanism by which increased mutation burden is attained. I find this incomprehensible with respect to any mechanism, including the ones cited in the manuscript (mutagenic by-products of estrogen metabolism or induced double-strand breaks). Could it be the case instead that higher mutation rates at ERBS set up the situation by which ER binding is enhanced (as you show in your data) and the accompanying increased expression of specific genes drives development of cancer? Here, knowing the comparative mutation burden for these constitutive ERBS and the corresponding expression levels of genes in normal breast cells might provide insights. It seems quite clear this possibility should be examined in your manuscript in order to (at least) rule it out. In other words, is it the ER activity that leads to the accumulation of somatic mutations or is it that somatic mutations at constitutive ERBS drives development of cancer because the enhanced binding and gene expression promotes uncontrolled cell growth and division?

**Are sufficient details provided to allow replication and comparison with related analyses that may have been performed?**

I feel that the details provided for various methods are quite complete, with the few confusing examples cited above that require more careful presentation. Other groups should be able to replicate these evaluations, assuming the corresponding data sets can be obtained. I think the overall approach is quite brilliant, and sets a very nice paradigm for advanced data integration that can be used to elucidate important mechanisms in cancer regarding non-coding recurrent mutations.

**Does the work represent a significant advance over previously published studies? Please explain.**

The advanced approaches to data integration used in this manuscript, to my knowledge, are novel and innovative and haven't been published previously. This manuscript provides a very nice approach to data integration across large data sets, aiming at a very important question about non-coding recurrent variants and how best to identify their functional consequences. The downstream Crispr experiments provide a beautiful validation of the approach and add impact to the conclusions arrived at from the data integration exercise.

**Is the paper of broad interest to others in the field, or of outstanding interest to a broad audience of biologists?**

Yes: The study represents an extensible example of data integration in large genomic data sets, of which there are increasing numbers, and permits others to pursue similar questions about other cancer genomics data.

Comments to author

My entire comments for the methods were too long to fit into the field, so they are copied in their entirety here:

The methods used in this study are appropriate for the aims, and represent a logical approach to addressing a very complex question about non-coding recurrent mutations in breast cancer genomes and their impact on the onset and progression of disease. The methods are reasonably well described, although there are several very confusing points in the text and in the figure legends that will need to be addressed in a revised version of the manuscript. In terms of controls, the only control missing from the analysis is one that provides information about where constitutive ER binding is occurring in normal breast cells. All the data utilized in these analyses is derived from breast cancer samples or breast cancer cell lines. Much interpretation about the mutations at ER binding sites is interpreted in the absence of a comparator normal data set for ER occupancy from ChIPseq. This proves ultimately to be a challenge for the resulting conclusions made by the authors, as detailed below.

Much of the confusion about methods stems from the lack of clear definition in the text about the different samples and data sets being used in the study. It would be advantageous to refer to the different data sets by a common identifier that would permit the reader to understand which samples are being referred to, rather than wondering which samples were, in fact, being discussed. Here is an example from top of page 11: "We detected the C to G conversion in five tumors whereas the G to C mutations in six patients; of these patients with the mutant alleles, two carry both mutations." In a single sentence, you have referred to tumors and patients and the meaning of anything from this sentence is subject to a lot of interpretation. In the figure legends, you must really do a better job of describing these very complex, multi-display figures and also tend to some fundamentals of data display, as follows:

Figure 2b. The box plot y-axis has no units on the expression levels of genes

Figure 2d. "Bar plot shows the number of patients carrying mutations at the top mutated ERBS" is really difficult to understand, and this gets further confusing when you have red bars in the bar plot AND red text to denote the two ERBS loci that you followed up on in your functional assays. Pick a different color, and make it clear the TEXT is in that color, not the bar, etc.

Figure 3c. data description in the legend is incredibly vague--really needs much more detail to interpret the blots and what is being identified from EMSA experiments.

Finally, the methods description of ChIA-PET based gene expression analysis (pg 31) needs to be re-written so it is understandable. At present, there are grammatical problems that make this obfuscated description in the first few sentences even more difficult to interpret.

**Authors Response**

**Point-by-point responses to the reviewers’ comments:**

Reviewer #1:

In this manuscript, Yang and colleagues studied overlap of somatic mutations in breast cancer with Estrogen Receptor binding events. An enrichment of somatic mutations at ER sites was observed, more specifically in those strong regions shared by multiple tumors, involved in 3D genome architecture. Using CRISPR, the authors altered specific ER sites, impacting 3D chromatin interactions (3C) and downstream gene expression. In general, I think this is an interesting paper, making intelligent use of many publicly available resources. Nonetheless, some results are in my opinion over-interpreted, and cause-effect relationships between ER binding and somatic mutations cannot be concluded given the data that is presented.

major comments:

1. It is stated in the manuscript, that specific enrichment of APOBEC-type mutations are observed at the ER sites. This is in line with work reported by Periyasamy et al., 2015 Cell Reports. This work should be acknowledged.

Answer: Thanks for the reminder. Periyasamy et al.’s work is one of the important references for us, but was missed in this statement. The citation is now added (page 7).

2. Multiple variables that are being reported in the study are actually linked, while this is presented as independent factors. Mutations are found at ER sites that shared by multiple tumors (Figure 1C), strongest sites (Figure 1B), and involved in ChIA-PET loops (Figure 2A). However, the strongest sites are always the ones shared by multiple tumors, and there is an intrinsic bias in the ChIA-PET data towards the strongest sites. Consequently, all these variables are linked and consequently not interpretable as separate entities. This could also very well explain the differential mutational frequency at the good and poor outcome ER sites (Figure 2C), and may thus represent an artefact.

Answer: We thank the reviewers for this great point. We agree with the reviewer that there are several interlinked measurements in our analysis in the first submission. Therefore, in light of the recommendations from this reviewer, we performed multiple additional analysis with the confounding factors controlled using negative binomial generalized linear regression models.

New figures have been added and corresponding text has been changed. Specifically, the current Supplementary Figure 1 shows ER binding sites shared by more patients contain more somatic mutations independent of their higher binding intensity (page 7). ER binding sites with more somatic mutations make more chromatin loop interactions, even after correcting for ER binding intensity (Figure 2a, Supplementary Figure 5; page 8). For the good and poor/metastasis outcome ER sites, we redid the analysis including ER binding intensity and number of chromatin interactions as covariates. Interestingly, the poor/metastasis-specific ER sites had significantly more mutations than the ER sites shared by more than 75% of all the samples (core ERBS) and the good outcome-specific ER sites (Figure 2c, Supplementary 6a; page 8-9). Corresponding part of the Methods section has been updated (page 33-34).

3. The ChIA-PET data as described in Figure 2 should be confirmed with Hi-C data. Also, the analyses in Figure 2B should be reperformed, taking TADs into consideration.

Answer: Thanks for the great suggestion. For the first part of this suggestion, limited by the resolution of the Hi-C data (40Kb is the highest resolution for MCF-7 Hi-C from Barutcu et al. 2015[1]), each Hi-C bin contains from 1 to 18 ERBS, as shown in the following figure. Thus unfortunately, the resolution of Hi-C hinders determination of the exact interacting sites for each ERBS.

For the second part of suggestion on the analysis in Figure 2b, taking TADs (40 kb resolution) detected in MCF-7 cells[1] into consideration further enhanced our original results as shown in the updated Figure 2b (page 8). Although the conclusion stays the same, corresponding P values became smaller.

4. It is stated in figure 4H that tamoxifen-induced cell death is blocked, but tamoxifen does not induce cell death at physiologically relevant concentrations, and the data shown in Figure 4H do not depict cell death, but colony growth instead; this is something completely different.

Answer: Thanks for the suggestion. We have changed all the statements of “tamoxifen-induced cell death” to “tamoxifen-induced growth inhibition” throughout the manuscript.

5. The introduction states that breast cancer is the most-common cancer related death. This is incorrect (lung cancer is; Ferlay et al., 2015), and this should be corrected.

Answer: Sorry for the confusion. We meant breast cancer causes the most number of cancer-related deaths in women. Ferlay et al. indicated 14.7% breast cancer deaths compared to 13.8% lung cancer deaths in women, which is in line with the claim. The citation for Ferlay et al., 2015 is added (page 4).

6. The hypothesis that forms the basis for the work, is that Estrogen Receptor chromatin interactions could play a causal role inducing somatic mutations. However, the current study does not address this hypothesis, as cause and effect are not separated with the current experimental design. With the current data, the authors did not convince this reviewer that ER binding would represent a mechanism of somatic mutation induction; it could simply represent an evolutionary pressure on stochastic events that ultimately alter the biological role of the main driver of the disease.

Answer: This is a great point. Thanks for the suggestion. We agree that to establish the causal relationship between ER and mutagenesis requires further experimental and computational verification. Our current data is not sufficient to rule out other possibilities. Thus we have modified the wording of our results and conclusions throughout the manuscript, and further discussed alternative possibilities as suggested by the reviewer. Although we still believe our results suggest causality, we agree that they are not definitive and thus we now better discuss alternative interpretations of the results in the Discussion section (page 14).

7. the analyses in Figure 4H indicate a specific somatic mutation to 'decrease tamoxifen-induced cell death (even though I don't agree with this, see point 4). As the patient samples used for somatic mutation analyses and the tumor samples used for ChIP-seq analyses were from treatment -naive patients, these tumors have not encountered tamoxifen yet. Does this represent a disconnect?

Answer: Thanks for the comment. The ChIP-seq tumor samples are indeed treatment-naïve. However, 99 out of the 560 patients with the WGS data had used or were using hormone therapy when the tissues were collected; out of the 99 patients, 51 have “TAM” or “Tamoxifen” listed in the donor therapy file provided by ICGC. Unfortunately, for the 5 patients carrying the C>T mutation at the ZNF143 locus, their chemotherapy treatment history is not well documented at the time of tissue collection. Specifically, two of the patients reported to have adjuvant radiation and unknown chemotherapy as their first and second therapies, respectively; two had unknown other therapy; and one was treatment-naïve. But we do not think this particular mutation was induced by tamoxifen treatment; instead, we believe it pre-existed to confer resistance to tamoxifen treatment. The existence of this mutation in the treatment-naïve patient and non-existence in the tamoxifen-treated patients partially supports our prediction. Yet we acknowledge that the current data does not enable us to draw any statistical conclusions (allele frequency of the mutation in the 560 patient cohort is ~0.45%; there are 0 mutant allele in the 99 patients with hormone therapy and 5 mutant alleles in the 461 patients either with unknown therapy or treatment-naive).

8. Are the 'good outcome' and 'poor outcome' Estrogen Receptor sites used in this study actually mutated in the samples that were used in the ChIP-seq analyses? As raw sequencing data for these ChIP-seq samples is available from the Ross-Innes paper, the authors should determine whether mutations are in fact associated with the differential ER binding in these very same samples. This is a critical point.

Answer: Thanks for the great point. We performed this analysis, and indeed identified mutations specific to ‘good outcome’ and ‘poor outcome’ ERBS. Because corresponding control tissues are not available for the 21 patients used in the Ross-Innes paper[2] and the original study design is ChIP-seq, we mapped reads covering the somatic mutation sites identified in the WGS study, and only included the loci with a sequencing depth of more than 10 reads for downstream analysis. Mutations passed these stringent criteria are more likely to be true somatic mutations in the ChIP-seq samples. The figure has been added (Supplementary Figure 6b). Corresponding Results and Materials and Methods sections have been modified accordingly (page 9, 35).

9. Is outcome data available for the patients for which WGS data was generated? Are somatic mutations at the observed sites also associated with outcome in these patients?

Answer: Thanks for the suggestion. We had tried to associate the mutation status with patients’ survival. However, only vital status (alive or deceased) was recorded for ~67% of all the patients in the WGS study. For the 377 patients with “alive” status, no follow-up status is available yet. The 7 patients with “deceased” status do not have survival-related data. There are 185 patients without vital status information. Thus unfortunately we are not able to explore the association between the particular mutations and corresponding patients’ survival. Moreover, we checked association between the mutations and age at diagnosis for patients, which we have more complete data on. Yet no association was found. This is expected based on our experimental results about the mutation at the ZNF143 locus, which does not affect cell proliferation but the response to tamoxifen.

10. In Figures 3 and 4, computational analyses are performed to show disrupted or created transcription factor binding motifs when a specific mutation is introduced. These data should be validated experimentally, by performing ChIP-QPCR analyses in the CRISPR models the authors have generated. This is a critical point.

Answer: Thanks for the suggestion. We have performed ChIP-qPCR at the mutation site for the ZNF143 locus. Indeed we saw a reduction of ZBTB7A binding at the particular site (Figure 4f), as suggested by our bioinformatics analysis and EMSA results. We used two different sets of primers to determine ZBTB7A enrichment in WT MCF-7 cells and one of the mutant clones used for other experiments in Figure 4. Two biological replicates of the experiment using another independent mutant clone also showed ZBTB7A binding reduction (Supplementary Figure 8). Main text has been updated to describe this new result (page 12). The protocol and agents used for the ChIP-qPCR experiment has been added to the Methods section (page 38-39).

11. As enhancer-activity is highly variable between tissue types, I feel it is not appropriate to use ChIP-seq data of cell lines from other tumor types to support the claims in the paper (Figure 4A).

Answer: We understand the reviewer’s concern. But the ChIP-qPCR results supported ZBTB7A binding at the locus. This suggested the binding site is conserved in K562, ISHIKAWA and MCF-7 cells. Thus we decided to keep the ZBTB7A ChIP-seq tracks from K562 and ISHIKAWA cells in Figure 4a.

12. the last part of the results section describing Figure 4 is quite elaborate, and the authors should consider moving parts to the discussion section.

Answer: We have moved parts from the Results section describing Figure 4 to the Discussion section (page 15-16). Thanks for the suggestion.

Reviewer #2: My entire comments for the methods were too long to fit into the field, so they are copied in their entirety here:

The methods used in this study are appropriate for the aims, and represent a logical approach to addressing a very complex question about non-coding recurrent mutations in breast cancer genomes and their impact on the onset and progression of disease. The methods are reasonably well described, although there are several very confusing points in the text and in the figure legends that will need to be addressed in a revised version of the manuscript. In terms of controls, the only control missing from the analysis is one that provides information about where constitutive ER binding is occurring in normal breast cells. All the data utilized in these analyses is derived from breast cancer samples or breast cancer cell lines. Much interpretation about the mutations at ER binding sites is interpreted in the absence of a comparator normal data set for ER occupancy from ChIPseq. This proves ultimately to be a challenge for the resulting conclusions made by the authors, as detailed below.

Answer: Thanks for the great suggestion. We tried our best to find constitutive ER binding sites from the literature, but did not have any luck. This data set is not available, possibly due to the difficulty of growing normal breast epithelium cells in vitro and performing ChIP-seq using primary normal tissues. We understand the importance of this data set for our data interpretation. Thus we changed the wording of our conclusion to make it more reflective of our current data and results. We also discussed three different models to interpret our data in the Discussion section (page 14) to further answer this reviewer’s question as detailed below.

Importantly, through a new set of analysis, we found that the mutation accumulation at ERBS is more significant (P = 7.20×10-32) in the 60 BRCA-EU patients using blood as “normal” for somatic mutation calling, but less significant (P = 2.18×10-3) in the 10 patients using tumor adjacent breast tissues as “normal” (Supplementary Figure 10). This suggests that ERBS-associated somatic mutations are likely to accumulate in normal/pre-neoplastic breast cells (page 15). We are in the process of designing and performing experiments to test the causal relationship and molecular mechanism underlying the coincidence of ER binding and higher than average mutation rates. However, these new experiments are beyond the scope of this manuscript.

Much of the confusion about methods stems from the lack of clear definition in the text about the different samples and data sets being used in the study. It would be advantageous to refer to the different data sets by a common identifier that would permit the reader to understand which samples are being referred to, rather than wondering which samples were, in fact, being discussed. Here is an example from top of page 11: "We detected the C to G conversion in five tumors whereas the G to C mutations in six patients; of these patients with the mutant alleles, two carry both mutations." In a single sentence, you have referred to tumors and patients and the meaning of anything from this sentence is subject to a lot of interpretation.

Answer: Thanks for the suggestion. Now we referred to the WGS data set as BRCA-EU and ChIP-seq data set as ER ChIP-seq throughout the manuscript. The dataset identifiers were defined clearly in the text (page 4). The sentence on the original page 11 (current page 9) was modified to clearly state the samples and data set.

In the figure legends, you must really do a better job of describing these very complex, multi-display figures and also tend to some fundamentals of data display, as follows:

Answer: Thanks for the critical reading. These suggestions improved our figure quality, and made the figures easily to understand by the readers.

Figure 2b. The box plot y-axis has no units on the expression levels of genes

Answer: We have now added the unit. The unit for gene expression is RSEM normalized count (Figure 2b).

Figure 2d. "Bar plot shows the number of patients carrying mutations at the top mutated ERBS" is really difficult to understand, and this gets further confusing when you have red bars in the bar plot AND red text to denote the two ERBS loci that you followed up on in your functional assays. Pick a different color, and make it clear the TEXT is in that color, not the bar, etc.

Answer: We have completely rewritten the figure legend for this plot. The two ERBS loci we followed up are now in bold instead of having confusing colors. The meaning of different colors for the bars and text fonts is explained in details in the figure legend (page 18-19).

Figure 3c. data description in the legend is incredibly vague--really needs much more detail to interpret the blots and what is being identified from EMSA experiments.

Answer: Figure 3c and 4c (EMSA results) are described in more details now (page 19-20). So the readers can better understand the experiments and interpret the results.

Finally, the methods description of ChIA-PET based gene expression analysis (pg 31) needs to be re-written so it is understandable. At present, there are grammatical problems that make this obfuscated description in the first few sentences even more difficult to interpret.

Answer: The corresponding Methods section has been rewritten (page 34). More technical details and new analysis were added to different parts of the Methods section.

--------------------

REFERENCES:

1.	Barutcu AR, Lajoie BR, McCord RP, Tye CE, Hong D, Messier TL, Browne G, van Wijnen AJ, Lian JB, Stein JL, et al: Chromatin interaction analysis reveals changes in small chromosome and telomere clustering between epithelial and breast cancer cells. Genome Biol 2015, 16:214.

2.	Ross-Innes CS, Stark R, Teschendorff AE, Holmes KA, Ali HR, Dunning MJ, Brown GD, Gojis O, Ellis IO, Green AR, et al: Differential oestrogen receptor binding is associated with clinical outcome in breast cancer. Nature 2012, 481:389-393.

**Second round of review**

**Reviewer 1**

most of my concerns have been addressed. However, two issues remained.

My comment #11 still feels inappropriately addressed however.

Minimally, the authors should acknowledge in the results or discussion section that K562 and ISHIKAWA are not suitable models to make statements on enhancer usage in breast cancer.

original question

11. As enhancer-activity is highly variable between tissue types, I feel it is not appropriate to use ChIP-seq data of cell lines from other tumor types to support the claims in the paper (Figure 4A).

Answer: We understand the reviewer’s concern. But the ChIP-qPCR results supported ZBTB7A binding at the locus. This suggested the binding site is conserved in K562, ISHIKAWA and MCF-7 cells. Thus we decided to keep the ZBTB7A ChIP-seq tracks from K562 and ISHIKAWA cells in Figure 4a.

For point 8, I would like to see the actual raw data, and variant calling files. How does the raw data look like, and how did the authors classify this as a variant? Figure S6 is very polished, and thus not transparently communicate the raw data anymore, making it impossible for me to assess the quality of the statement and solidity of the conclusions.

original question

8. Are the 'good outcome' and 'poor outcome' Estrogen Receptor sites used in this study actually mutated in the samples that were used in the ChIP-seq analyses? As raw sequencing data for these ChIP-seq samples is available from the Ross-Innes paper, the authors should determine whether mutations are in fact associated with the differential ER binding in these very same samples. This is a critical point.

Answer: Thanks for the great point. We performed this analysis, and indeed identified mutations specific to ‘good outcome’ and ‘poor outcome’ ERBS. Because corresponding control tissues are not available for the 21 patients used in the Ross-Innes paper[2] and the original study design is ChIP-seq, we mapped reads covering the somatic mutation sites identified in the WGS study, and only included the loci with a sequencing depth of more than 10 reads for downstream analysis. Mutations passed these stringent criteria are more likely to be true somatic mutations in the ChIP-seq samples. The figure has been added (Supplementary Figure 6b). Corresponding Results and Materials and Methods sections have been modified accordingly (page 9, 35).

**Reviewer 2**

The manuscript improved in many ways based on comments from referees. The figures are tremendously better, the attempts to address certain concerns are fine but in some cases were perhaps written a bit hastily so they require significant clarification by rewriting. My primary concern remains, namely that the authors have presented compelling data but data that only suggest discrete hypotheses, none of which is confirmed by the data in-hand. As such, the findings absolutely have merit but are at risk of being over-interpreted. Good science always begets more experiments, so I do not see this as a foundational flaw that not all the questions are answered and new ones are raised. However, I do think the conclusions must be softened so the remaining questions are clear and clearly acknowledged. Finally while this may look a bit of a laundry list, I went through the paper extensively so the language could be clarified where needed. This will only result in a more readable paper, in my opinion, that showcases the science as it should be.

My comments, for consideration by the authors, follow herein:

Pg2, line 11: State explicitly that you are focused on ‘two of the recurrently mutated ERBS sites’

Is ZNF143 mutation associated with decreased TF binding or ER binding??

Final sentence in the abstract needs to be softened.

pg 4, line 12-14: This is poorly written. "ER binding activity" is not the major transcription factor mediating estrogen response in breast cancer. ER is. I think this sentence, even though poorly written, is deterministic. Softening it to state that you hypothesized there might be a mechanistic link between ER and recurrent somatic mutations in non-coding DNA in ER positive breast cancer is perhaps better.

Line 18: If you are going to state the analysis shows a significant result “significant accumulation” then you need a p value. I am also not sure “accumulation” is the correct term to use here. ‘Proportion’ is probably more accurate.

Line 20: Isn’t this sentence (“We also identified multiple novel recurrent non-coding mutations at ERBS”) redundant with all the information before it? If not, provide a better definition/word than “novel” to distinguish this sentence from other information presented. Do you mean "unique"?

Page 5, line 2: Do you mean “ER” instead of “TF” here? If yes, please replace for accuracy and clarity.

Line 8: You are not investigating ‘increased rates of mutations’, rather ‘increased mutational frequency’ at ERBS is more accurate.

Line 9: This sentence is really awkward. Rather than ‘acquired ER binding events’, it would be more clear to state: “We acquired ER binding profiles from Ross-Innes et al. (ER ChIP-seq data) from eight good outcome ER+, etc.”

Page 7, line 1: “Intensity” of ER binding is probably not the right term. It seems your analysis is of constitutive versus non-constitutive binding, so should this be called ‘occupancy’?. This raises the question of where these data are obtained from? This is not stated clearly and should be added so it clarifies what feature you are evaluating for different ERBS.

Page 9, line 21/22: Does “TF” here mean ER? Or are you examining all known motifs for all TFs? Please clarify. What is “MAX TF”? It’s not been defined yet in the manuscript. Also, please find a more definitive descriptor than “the most” to describe what you are looking at in terms of disruption and how it is quantified.

Pg 12: For the ZBTB7A results that are described, how are these relevant to ER binding? Is the ZBTB7A binding locus also an ERBS? Please clarify in text.

Pg 14, Line 5&6: Your citation and text disagree with one another. Paper #50 is from 2015 and so is not from 3 decades ago, nor does it present data to show that estradiol is a mutagen. That appears to be paper 52, which is from the year 2000 (also not three decades ago).

Pg 14: Appreciate the expanded hypotheses that might explain your results. These are quite poorly written in the revision, however and need to be addressed to avoid confusion. You seem to suggest that, 1) constitutively bound ERBS are not readily repaired and hence the proportion of somatic alterations becomes skewed due to protection of the DNA OR 2) that selection (not ER binding per se) of these mutations in the tumor development from normal cells occurs by reprogramming of ER binding and gene expression changes, OR 3) ER binding/transcription directly causes somatic mutations at ERBS. These are all fine hypotheses. I don’t understand how your results support the latter hypothesis, however. You haven’t shown a mechanism so it’s not appropriate to declare victory at this point. Your data do not demonstrate DNA ds breaks result from ER binding at all, nor do they suggest a mechanism. Hypothesis and mechanism 1 are supported by reports in the literature that you cite (48&49). Hypothesis and mechanism 2, however, are supported by your data with the caveats that your functional studies do not directly imply ER binding but rather are focused on non-ER TFs that apparently also bind at ERBS (this is the best interpretation I can make based on the questions raised earlier in the review).

**Authors Response**

**Point-by-point responses to the reviewers’ comments:**

Reviewer #1:

most of my concerns have been addressed. However, two issues remained.

My comment #11 still feels inappropriately addressed however.

Minimally, the authors should acknowledge in the results or discussion section that K562 and ISHIKAWA are not suitable models to make statements on enhancer usage in breast cancer.

New Answer: Thanks for the comments. We have changed the sentence and added the acknowledgement in the Results section (page 11, highlighted). Specifically, we added the following sentence to the text, “ZBTB7A ChIP-seq data is not available in breast cancer cell lines. However, although TF binding is context and cell line-specific, the publicly available data for K562 leukemia and Ishikawa endometrial cancer cells show strong ZBTB7A binding to this site”.

original question

11. As enhancer-activity is highly variable between tissue types, I feel it is not appropriate to use ChIP-seq data of cell lines from other tumor types to support the claims in the paper (Figure 4A).

Answer: We understand the reviewer’s concern. But the ChIP-qPCR results supported ZBTB7A binding at the locus. This suggested the binding site is conserved in K562, ISHIKAWA and MCF-7 cells. Thus we decided to keep the ZBTB7A ChIP-seq tracks from K562 and ISHIKAWA cells in Figure 4a.

For point 8, I would like to see the actual raw data, and variant calling files. How does the raw data look like, and how did the authors classify this as a variant? Figure S6 is very polished, and thus not transparently communicate the raw data anymore, making it impossible for me to assess the quality of the statement and solidity of the conclusions.

New Answer: We appreciate the Reviewer’s efforts to make each statement reliable. For calling the variants, firstly we selected the genomic positions both mutated in the BRCA-EU WGS study and within the good/poor outcome ERBS. Using the BRCA-EU mutation sites is due to the lack of proper controls in the ER ChIP-seq data. We hypothesize that genomic positions mutated in the 560 BRCA-EU WGS samples are more likely to be mutated in the ER ChIP-seq samples. Then we used merged bam files of the outcome-specific ER ChIP-seq samples to map alleles at these selected positions. Only the genomic positions with more than 10 sequencing reads were retained for variant calling (the variant annotation files after this step were attached as Supplementary Data 1 & 2; page 24). The genomic positions with at least 1 read for the alternative allele were defined as potentially mutated. Figure S6 plots the percentage of ERBS with at least one potential somatic mutation for good/poor outcome-associated ERBS, respectively.

original question

8. Are the 'good outcome' and 'poor outcome' Estrogen Receptor sites used in this study actually mutated in the samples that were used in the ChIP-seq analyses? As raw sequencing data for these ChIP-seq samples is available from the Ross-Innes paper, the authors should determine whether mutations are in fact associated with the differential ER binding in these very same samples. This is a critical point.

Answer: Thanks for the great point. We performed this analysis, and indeed identified mutations specific to ‘good outcome’ and ‘poor outcome’ ERBS. Because corresponding control tissues are not available for the 21 patients used in the Ross-Innes paper[2] and the original study design is ChIP-seq, we mapped reads covering the somatic mutation sites identified in the WGS study, and only included the loci with a sequencing depth of more than 10 reads for downstream analysis. Mutations passed these stringent criteria are more likely to be true somatic mutations in the ChIP-seq samples. The figure has been added (Supplementary Figure 6b). Corresponding Results and Materials and Methods sections have been modified accordingly (page 9, 35).

Reviewer #2:

The manuscript improved in many ways based on comments from referees. The figures are tremendously better, the attempts to address certain concerns are fine but in some cases were perhaps written a bit hastily so they require significant clarification by rewriting. My primary concern remains, namely that the authors have presented compelling data but data that only suggest discrete hypotheses, none of which is confirmed by the data in-hand. As such, the findings absolutely have merit but are at risk of being over-interpreted. Good science always begets more experiments, so I do not see this as a foundational flaw that not all the questions are answered and new ones are raised. However, I do think the conclusions must be softened so the remaining questions are clear and clearly acknowledged. Finally while this may look a bit of a laundry list, I went through the paper extensively so the language could be clarified where needed. This will only result in a more readable paper, in my opinion, that showcases the science as it should be.

New Answer: The reviewer’s extensive reading and help with wording clarification is highly appreciated. We have read the reviewer’s comments carefully. We agree with most of the recommendations from this reviewer and made necessary changes.

My comments, for consideration by the authors, follow herein:

Pg2, line 11: State explicitly that you are focused on ‘two of the recurrently mutated ERBS sites’

New Answer: Changed. Specifically, we wrote “we focused on two of the recurrently mutated ERBS”.

Is ZNF143 mutation associated with decreased TF binding or ER binding??

New Answer: The mutation is 125 bps away from the ERBS. Our computational analysis suggested that the mutation disrupts the DNA binding motif of ZBTB7A TF. In line with this, our experimental ChIP-qPCR data shows that the C→T mutation at the ZNF143 locus results in decreased binding activity of ZBTB7A.

Final sentence in the abstract needs to be softened.

New Answer: We believe the data presented in this manuscript is in line with our conclusion in the last sentence, which states “ER binding is associated with localized accumulation of somatic mutations, some of which affect chromatin architecture, distal gene expression and cellular phenotypes in ER positive breast cancer”.

pg 4, line 12-14: This is poorly written. "ER binding activity" is not the major transcription factor mediating estrogen response in breast cancer. ER is. I think this sentence, even though poorly written, is deterministic. Softening it to state that you hypothesized there might be a mechanistic link between ER and recurrent somatic mutations in non-coding DNA in ER positive breast cancer is perhaps better.

New Answer: Changed. We specifically wrote “we hypothesized that there might be a mechanistic link between ER, the major transcription factor (TF) mediating estrogen response in breast cancer, and localized non-coding mutational load in the ER-positive breast cancer genome”.

Line 18: If you are going to state the analysis shows a significant result “significant accumulation” then you need a p value. I am also not sure “accumulation” is the correct term to use here. ‘Proportion’ is probably more accurate.

New Answer: Changed. We specifically wrote “The integrative analysis shows a disproportionately large amount of somatic mutations at ER binding sites (ERBS)”.

Line 20: Isn’t this sentence (“We also identified multiple novel recurrent non-coding mutations at ERBS”) redundant with all the information before it? If not, provide a better definition/word than “novel” to distinguish this sentence from other information presented. Do you mean "unique"?

New Answer: Sorry for the confusion. We changed “novel” to “uncharacterized”, and defined “recurrent” as “existing in more than one patient”.

Page 5, line 2: Do you mean “ER” instead of “TF” here? If yes, please replace for accuracy and clarity.

New Answer: We mean TFs such as ZBTB7A rather than ER here. We explicitly stated it in the revision.

Line 8: You are not investigating ‘increased rates of mutations’, rather ‘increased mutational frequency’ at ERBS is more accurate.

New Answer: We appreciate the recommendation. It is edited accordingly.

Line 9: This sentence is really awkward. Rather than ‘acquired ER binding events’, it would be more clear to state: “We acquired ER binding profiles from Ross-Innes et al. (ER ChIP-seq data) from eight good outcome ER+, etc.”

New Answer: We appreciate the recommendation. It is edited accordingly.

Page 7, line 1: “Intensity” of ER binding is probably not the right term. It seems your analysis is of constitutive versus non-constitutive binding, so should this be called ‘occupancy’?. This raises the question of where these data are obtained from? This is not stated clearly and should be added so it clarifies what feature you are evaluating for different ERBS.

New Answer: Sorry for the confusion. We included two different types of analysis in the first part of this paragraph: 1) ER binding intensity, and 2) ER occupancy. Both features are extracted from ER ChIP-seq data. Clarification of the data used was added.

Page 9, line 21/22: Does “TF” here mean ER? Or are you examining all known motifs for all TFs? Please clarify. What is “MAX TF”? It’s not been defined yet in the manuscript. Also, please find a more definitive descriptor than “the most” to describe what you are looking at in terms of disruption and how it is quantified.

New Answer: Sorry for the confusion. We are examining known motifs of all TFs here. “MAX TF” was changed to MYC Associated Factor X (MAX). For the measurement, we are quantifying the probabilities of all known motifs in genomic sequences with and without the mutation. If the probability is decreased with the presence of the mutation, the motif is predicted to be disrupted. Conversely, if the probability is increased, a novel motif is predicted to be created. Corresponding sentences have been modified to specify the measurement.

Pg 12: For the ZBTB7A results that are described, how are these relevant to ER binding? Is the ZBTB7A binding locus also an ERBS? Please clarify in text.

New Answer: Thanks for the suggestion. The ZBTB7A binding is 125 bps upstream of ER binding summit in the ER ChIP-seq data. How the decreased binding of ZBTB7A affects ER binding is unknown. The clarification is added in text.

Pg 14, Line 5&6: Your citation and text disagree with one another. Paper #50 is from 2015 and so is not from 3 decades ago, nor does it present data to show that estradiol is a mutagen. That appears to be paper 52, which is from the year 2000 (also not three decades ago).

New Answer: We apologize for the mistake and appreciate the careful analysis. The final references were attached after the Materials and Methods section in the last submission. The references after the Discussion section were incomplete, which has been deleted now.

Pg 14: Appreciate the expanded hypotheses that might explain your results. These are quite poorly written in the revision, however and need to be addressed to avoid confusion. You seem to suggest that, 1) constitutively bound ERBS are not readily repaired and hence the proportion of somatic alterations becomes skewed due to protection of the DNA OR 2) that selection (not ER binding per se) of these mutations in the tumor development from normal cells occurs by reprogramming of ER binding and gene expression changes, OR 3) ER binding/transcription directly causes somatic mutations at ERBS. These are all fine hypotheses. I don’t understand how your results support the latter hypothesis, however. You haven’t shown a mechanism so it’s not appropriate to declare victory at this point. Your data do not demonstrate DNA ds breaks result from ER binding at all, nor do they suggest a mechanism. Hypothesis and mechanism 1 are supported by reports in the literature that you cite (48&49). Hypothesis and mechanism 2, however, are supported by your data with the caveats that your functional studies do not directly imply ER binding but rather are focused on non-ER TFs that apparently also bind at ERBS (this is the best interpretation I can make based on the questions raised earlier in the review).

New Answer: We really appreciate the great insights by this reviewer. After carefully re-evaluating and thinking about our interpretations of the data and results, we largely agree with the Reviewer’s comment. We therefore edited the paragraph accordingly (pages 14-15, highlighted).

1.	Hu M, Yu J, Taylor JM, Chinnaiyan AM, Qin ZS: On the detection and refinement of transcription factor binding sites using ChIP-Seq data. Nucleic Acids Res 2010, 38:2154-2167.
